# CD4^+^ T cells promote humoral immunity and viral control during Zika virus infection

**DOI:** 10.1371/journal.ppat.1007474

**Published:** 2019-01-24

**Authors:** Annie Elong Ngono, Matthew P. Young, Maximilian Bunz, Zhigang Xu, Sararat Hattakam, Edward Vizcarra, Jose Angel Regla-Nava, William W. Tang, Montarop Yamabhai, Jinsheng Wen, Sujan Shresta

**Affiliations:** 1 Division of Inflammation Biology, La Jolla Institute for Allergy and Immunology, La Jolla, CA, United States of America; 2 Institute of Arboviruses, School of Basic Medical Sciences, Wenzhou Medical University, Wenzhou, Zhejiang, China; 3 School of Biotechnology, Institute of Agricultural Technology, Suranaree University of Technology, Nakhon Ratchasima, Thailand; 4 Department of Medicine, School of Medicine, University of California San Diego, La Jolla, CA, United States of America; Icahn School of Medicine at Mount Sinai, UNITED STATES

## Abstract

Several Zika virus (ZIKV) vaccines designed to elicit protective antibody (Ab) responses are currently under rapid development, but the underlying mechanisms that control the magnitude and quality of the Ab response remain unclear. Here, we investigated the CD4^+^ T cell response to primary intravenous and intravaginal infection with ZIKV. Using the *LysMCre*^*+*^*Ifnar1*^*fl/fl*^ (myeloid type I IFN receptor-deficient) C57BL/6 mouse models, we identified six I-A^b^-restricted ZIKV epitopes that stimulated CD4^+^ T cells with a predominantly cytotoxic Th1 phenotype in mice primed with ZIKV. Intravenous and intravaginal infection with ZIKV effectively induced follicular helper and regulatory CD4^+^ T cells. Treatment of mice with a CD4^+^ T cell-depleting Ab reduced the plasma cell, germinal center B cell, and IgG responses to ZIKV without affecting the CD8^+^ T cell response. CD4^+^ T cells were required to protect mice from a lethal dose of ZIKV after infection intravaginally, but not intravenously. However, adoptive transfer and peptide immunization experiments showed a role for memory CD4^+^ T cells in ZIKV clearance in mice challenged intravenously. These results demonstrate that CD4^+^ T cells are required mainly for the generation of a ZIKV-specific humoral response but not for an efficient CD8^+^ T cell response. Thus, CD4^+^ T cells could be important mediators of protection against ZIKV, depending on the infection or vaccination context.

## Author summary

Several vaccine candidates are currently under development for Zika virus (ZIKV) infection, which causes life-threatening neurologic diseases, including congenital Zika syndrome in neonates and Guillain-Barré syndrome in adults. However, the mechanisms by which the adaptive immune system contributes to protection against ZIKV have not been fully investigated. In this report, we used mouse models of systemic and genital mucosal infection with ZIKV to evaluate the role of CD4^+^ T cells in regulating antiviral humoral and CD8^+^ T cell responses and in controlling ZIKV infection. We first defined the antigen specificity and phenotype of the CD4^+^ T cell response to ZIKV and then demonstrated that CD4^+^ T cells are required for inducing humoral, but not CD8^+^ T cell, responses in ZIKV-primed mice. CD4^+^ T cells were also required for local control of ZIKV burden in mice infected intravaginally. Although CD4^+^ T cells are not necessary for the control of primary ZIKV infection via the intravenous route, CD4^+^ T cells can confer protection against lethal intravaginal ZIKV challenge. Our results support ZIKV vaccine strategies that induce a protective CD4^+^ T cell response to ZIKV.

## Introduction

Research on the immune response to infection with Zika virus (ZIKV), a member of the *Flaviviridae* family that includes dengue virus (DENV), yellow fever virus (YFV), and West Nile virus (WNV), has intensified since the most recent outbreak in 2015 in Brazil. Many flavivirus infections are transmitted through the bite of infected mosquitoes. However, ZIKV shows some critical features in its transmission routes and clinical outcomes. ZIKV can be transmitted via sexual contact [1, 2], persists for weeks in the reproductive tract [3–5], and undergoes vertical transmission from mother to fetus [6–8]. ZIKV infection of pregnant women has been associated with an increased incidence of congenital disorders in fetuses, including microcephaly [9], whereas ZIKV infection of adults is linked to the neurological disorder Guillain-Barré syndrome [10]. Given the range of clinical symptoms, there is a pressing need to understand how different transmission routes affect the immune response to ZIKV infection.

Accumulating evidence suggests that both cellular and humoral responses are required for effective control of ZIKV [11]. Infection with ZIKV induces the production of neutralizing antibodies (Abs), as evidenced by a study of two independent patient cohorts from Brazil and Mexico, where ZIKV is endemic [12]. In rhesus macaques, primary ZIKV infection induces neutralizing Abs that may be important for control of viral replication [13], and the production of neutralizing Abs correlates with protection against secondary ZIKV infection [14]. Several groups have demonstrated in mouse models that protection against ZIKV can be conferred by a variety of monoclonal Abs, including DENV/ZIKV-cross-reactive Abs, and by vaccine-induced Ab responses [15–21].

Compared with the humoral response, relatively little is known about the cellular immune response to ZIKV, especially CD4^+^ T cell responses. We and others recently identified an important role for CD8^+^ T cells in controlling ZIKV infection using H-2^b^ mouse models [22, 23]. In H-2^b^ mice, CD8^+^ T cells targeted peptides from all ZIKV proteins (three structural proteins [Capsid, Pre-membrane, Envelope] and seven nonstructural proteins [NS1, NS2A, NS2B, NS3, NS4A, NS4B, NS5]), with a preference for the structural proteins [22]. In addition, DENV/ZIKV cross-reactive CD8^+^ T cells played an important role in protecting against ZIKV in peptide vaccination and sequential DENV-ZIKV infection settings in various mice, including HLA transgenic and pregnant animals [24–26].

Studies of mouse models of flaviviral infection, including WNV [27], DENV [28], and YFV 17D [29], have suggested that CD4^+^ T cells, particularly Th1 subsets, contribute to protection against infection. Accordingly, Pardy and colleagues revealed that CD4^+^ T cells responding to ZIKV infection in wildtype mice were also predominantly of a Th1 phenotype, although the response to isolated ZIKV peptides was not investigated in this study [30]. In another report, Winkler and colleagues detected proliferation of CD4^+^ T cells in response to ZIKV infection of wildtype mice [31]. However, the role of CD4^+^ T cells in generating efficient anti-ZIKV humoral and cellular responses and in mediating protection remains unclear, as does the extent to which CD4^+^ T cell subsets, such as follicular helper T (T_FH_) cells and regulatory T (Treg) cells, are involved in the response. Because a variety of ZIKV vaccine candidates are under accelerated development, it is important to understand the precise contribution of CD4^+^ T cells to protection against ZIKV and to determine whether ZIKV vaccine designs should optimize CD4^+^ T cell responses.

In this study, we investigated the role of CD4^+^ T cells in the response to primary ZIKV infection via systemic (intravenous) and sexually transmitted (intravaginal) routes using *LysMCre*^*+*^*Ifnar1*^*fl/fl*^ and *Ifnar1*^*-/-*^ C57BL/6 mice, as we described previously for investigation of the CD8^+^ T cell response to ZIKV [22]. We evaluated the immune response via the two infection routes with respect to: the immunodominant H-2^b^-restricted ZIKV epitopes, the quality and kinetics of activation of CD4^+^ T cell subsets, the requirement for CD4^+^ T cells in inducing ZIKV-specific Ab and CD8^+^ T cell responses, and the impact on viral clearance. We found that the CD4^+^ T cell response to primary infection was predominantly Th1 and was directed against a narrow range of immunodominant ZIKV epitopes in E, NS3, NS4B, and NS5 proteins. Notably, CD4^+^ T cells contributed to the ZIKV-specific plasma cell, germinal center (GC) B cell, and IgG responses after both intravenous and intravaginal infection. However, CD4^+^ T cells were required for local control of viral infection in the lower female reproductive tract and for protection against lethal intravaginal ZIKV infection. Additionally, memory CD4^+^ T cells contributed to viral clearance in mice after primary, but not secondary, intravenous ZIKV infection. Our data suggest that efficient ZIKV vaccines should promote CD4^+^ T cell activation.

## Results

### Predominant Th1 response to primary ZIKV infection via the intravenous route in *LysMCre*^*+*^*Ifnar1*^*fl/fl*^ mice

**Fig 1:**
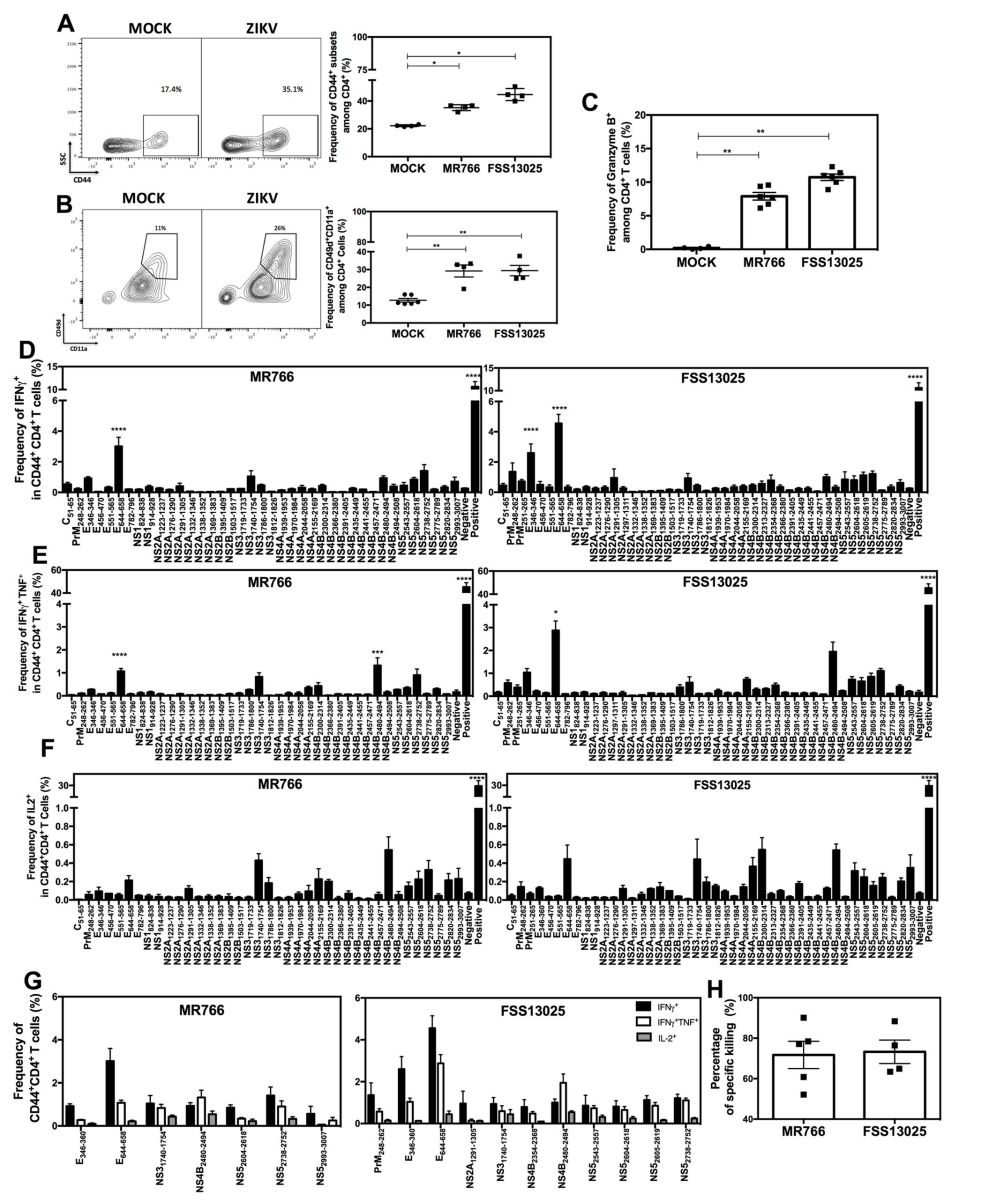
Mapping of the CD4^+^ T cell response in the *LysMCre*^+^*Ifnar1*^fl/fl^ mouse model of primary ZIKV infection. Five-week-old *LysMCre*^+^*Ifnar1*^fl/fl^ C57BL/6 mice were infected retro-orbitally with 10^4^ FFU of ZIKV strain MR766 or FSS13025 in 10% FBS/PBS or were mock-infected (10% FBS/PBS alone). Data are the mean ± SEM of *n* = 4–6 mice/group. (**A–C**) Splenocytes were removed on day 7 post-infection and analyzed for the percentage of (**A**) CD44^+^CD4^+^ T cells, (**B**) CD49d^+^CD11a^+^ T cells, and (**C**) granzyme B^+^CD4^+^ T cells. (**D–F**) Splenocytes were removed on day 7 post-infection and stimulated with the indicated ZIKV-derived peptides and brefeldin A. The percentage CD44^+^CD4^+^ T cells producing (**D**) IFNγ, (**E**) IFNγ and TNF, and (**F**) IL-2 was measured by ICS. Cells stimulated with DMSO or PMA/ionomycin served as negative and positive controls, respectively. (**G**) Summary of the data shown in (**D–F**). (**H**) *In vivo* killing of ZIKV peptide-pulsed target cells. *LysMCre*^+^*Ifnar1*^fl/fl^ mice were retro-orbitally mock-infected (*n* = 4) or infected with 10^4^ FFU ZIKV MR766 (*n* = 5) and FSS13025 (*n* = 4) for 7 days, and then injected retro-orbitally with naïve C57BL/6 splenocytes (*n* = 4) pulsed with a pool of ZIKV peptides (E_346-360,_ E_644-658_, NS3_1740-1754_, NS4B_2480-2494_, NS5_2604-2618_, NS5_2738-2752_) or treated with DMSO. After 12 h, the splenocytes were harvested from recipient mice, analyzed by flow cytometry, and the percentage ZIKV-specific cytotoxicity was calculated. *P < 0.05, **P < 0.01 by the Mann–Whitney *U* test. The production of cytokines after stimulation with each peptide was compared to the negative control (DMSO) using one-way ANOVA t-test. **** *P* < 0.0001. Data are representative of two independent experiments.

We have previously employed the *LysMCre*^*+*^*Ifnar1*^*fl/fl*^ C57BL/6 mouse model to study the response of CD8^+^ T cells to ZIKV infection [22]. To validate the model for investigation of CD4^+^ T cells, 5-week-old *LysMCre*^*+*^*Ifnar1*^*fl/fl*^ mice were intravenously (retro-orbitally, RO) infected with ZIKV strains MR766 or FSS13025 or were mock-infected with vehicle (10% FBS/PBS). Splenocytes were prepared 7 days later and analyzed for activated (CD44^+^) or antigen-experienced (CD49d^+^CD11a^+^) CD4^+^ T cells by flow cytometry. We found that cells from ZIKV-infected mice contained significantly more (2-fold) activated and antigen-experienced CD4^+^ T cells compared with cells from mock-infected mice (**Fig 1A and 1B**). We also noted a striking increase in the number of CD4^+^ T cells with cytotoxic potential (granzyme B^+^) in the spleens of ZIKV-infected mice (**Fig 1C**), which is consistent with the known contribution of cytotoxic effector CD4^+^ T cells in anti-flaviviral control [27, 32]. These results confirmed the suitability of *LysMCre*^*+*^*Ifnar1*^*fl/fl*^ mice to study the CD4^+^ T cell response to ZIKV infection.

To identify potential CD4^+^ T cell epitopes in the ZIKV proteome, we screened the Immune Epitope Data Base for predicted class II (H-2 I-A^b^)-binding epitopes and selected 49 peptides (the top 1% of candidates) for further testing *in vitro* (**Table 1**). Splenocytes were harvested from ZIKV- or mock-infected *LysMCre*^*+*^*Ifnar1*^*fl/fl*^ on day 7 post-infection and stimulated with the individual peptides. Flow cytometric intracellular staining (ICS) assays were then performed to determine the frequency of CD44^+^CD4^+^ T cells producing Th1 (IFNγ, TNF, IL-2), Th2 (IL-4, IL-5), or Th17 (IL-17A) cytokines. A strong Th1 response, as indicated by the production of IFNγ (with or without TNF) and IL-2, was induced by both the African and Asian ZIKV strain (**Fig 1D–1F**). Six immunodominant epitopes derived from the structural E protein (E_346-360_, E_644-658_) and nonstructural NS3 (NS3_1740-1754_), NS4B (NS4B_2480-2494_), and NS5 (NS5_2604-2618_, NS5_2738-2752_) proteins stimulated a particularly vigorous response by both ZIKV MR766- and FSS13025-primed CD4^+^ T cells (**Fig 1D–1G**). However, none of the immunodominant epitopes stimulated the production of Th2 or Th17 cytokines (**S1A–S1C Fig**), confirming the primacy of the Th1 response.

**Table 1:** Summary of predicted ZIKV-derived CD4^+^ T cell epitopes.

Sequence	Length	I-Ab	MR766	FSS13025	Protein	Start_position	End_position
AAAIFMTATPPGTRD	15	X	X	X	NS3	1812	1826
AALTTFITPAVQHAV	15	X		X	NS4B	2313	2327
ALAILAALTPLARGT	15	X	X	X	NS2A	1291	1305
DIDLRPASAWAIYAA	15	X	X	X	NS4B	2300	2314
DRYKYHPDSPRRLAA	15	X	X	X	NS1	824	838
EDVNLGSGTRAVASC	15	X	X	X	NS5	2775	2789
EFYSYKKSGITEVCR	15	X	X	X	NS4B	2543	2557
EMYWVSGAKSNIIKS	15	X	X		NS5	2738	2752
**EMYWVSGAKSNTIKS**	**15**	**X**		**X**	**NS5**	**2738**	**2752**
ENWIFRNPGFALVAV	15	X	X	X	PrM	248	262
GDEYMYGGGCAETDE	15	X	X	X	NS3	1970	1984
GERVILAGPMPVTHA	15	X	X	X	NS3	1939	1953
GGVLIFLSTAVSADV	15	X		X	E	782	796
GGVMIFLSTAVSADV	15	X	X		E	782	796
GGWSYYAATIRKVQE	15	X		X	NS4B	2605	2619
**GLPVRYMTTAVNVTH**	**15**	**X**	**X**	**X**	**NS3**	**1740**	**1754**
IFRNPGFALAAAAIA	15	X		X	M	251	265
KADIEMAGPMAAVGL	15	X	X		NS2B	1395	1409
KKNLPFVAALGLTAV	15	X	X		NS2A	1338	1352
KKNLPFVMALGLTAV	15	X		X	NS2A	1338	1352
KSYFVRAAKTNNSFV	15	X	X		NS1	914	928
KVEITPNSPRAEATL	15	X		X	E	456	470
KVEVTPNSPRAEATL	15	X	X		E	456	470
LAFLRFTAIKPSLGL	15	X	X	X	C	51	65
LALVAAFKVRPALLV	15	X	X	X	NS2A	1223	1237
LRTVILAPTRVVAAE	15	X	X	X	NS3	1719	1733
MDEAHFTDPSSIAAR	15	X	X	X	NS3	1786	1800
MIGCYSQLTPLTLIV	15	X		X	NS4B	2366	2380
MMGCYSQLTPLTLIV	15	X	X		NS4B	2366	2380
NGFALAWLAIRAMAV	15	X	X		NS2A	1276	1290
NGFALAWLAIRAMVV	15	X		X	NS2A	1276	1290
NIFRGSYLAGASLIY	15	X	X		NS4B	2494	2508
PFYAWDFGVPLLMIG	15	X		X	NS4B	2354	2368
**PNKYWNSSTATSLCN**	**15**	**X**	**X**	**X**	**NS4B**	**2480**	**2494**
**PVGRLITANPVITES**	**15**	**X**	**X**	**X**	**E**	**644**	**658**
PVWLAYQVASAGITY	15	X	X	X	NS4A	2044	2058
PYRTWAYHGSYEAPT	15	X	X	X	NS5	2820	2834
QEGAVHTALAGALEA	15	X	X	X	E	551	565
QVLLIAVAISSAVLL	15	X	X		NS4B	2441	2455
QVLLIAVAVSSAILS	15	X		X	NS4B	2441	2455
RAIWYMWLGARFLEF	15	X	X	X	NS5	2993	3007
**RGGWSYYAATIRKVQ**	**15**	**X**	**X**	**X**	**NS5**	**2604**	**2618**
SGALWDVPAPKEVKK	15	X	X	X	NS2B	1503	1517
SGKRSWPPSEVLTAV	15	X	X	X	NS2A	1369	1383
TAWGWGEAGALITAA	15	X	X	X	NS4B	2457	2471
TGSRPYKAAAAQLPE	15	X	X	X	NS4A	2155	2169
TLAILAALTPLARGT	15	X		X	NS2A	1297	1311
**VRSYCYEASISDMAS**	**15**	**X**	**X**	**X**	**E**	**346**	**360**
YLIPGLQAAAARAAQ	15	X	X	X	NS4B	2391	2405

The sequence, length and position of peptides from MR766 and FSS13025 ZIKV strains are represented.

We confirmed that the granzyme B^+^CD44^+^CD4^+^ T cells detected *in vitro* were *bona fide* cytolytic cells by performing an *in vivo* cytotoxicity assay. Naïve C57BL/6 splenocytes (CD45.1) were incubated with medium alone or pulsed with a mixture of the six immunodominant ZIKV epitopes and then labeled with a low (control target cells) or high (antigen-specific target cells) concentration of CSFE. The target cells were mixed and injected into mock- or ZIKV-infected mice, and splenocytes were harvested after 12 h and examined by flow cytometry to quantify low- and high-CSFE cells. As shown in **Fig 1H**, we detected approximately 70% specific killing of ZIKV epitope-pulsed target cells by both MR766- and FSS13025-primed mice. Taken together, these results indicate that Asian and African ZIKV strains both induce a robust expansion of cytokine-secreting and cytotoxic Th1 effector CD4^+^ T cells in *LysMCre*^*+*^*Ifnar1*^*fl/fl*^ mice.

### Expansion of follicular helper CD4^+^ T cells and reduction of regulatory CD4^+^ T cells during primary ZIKV infection

**Fig 2:**
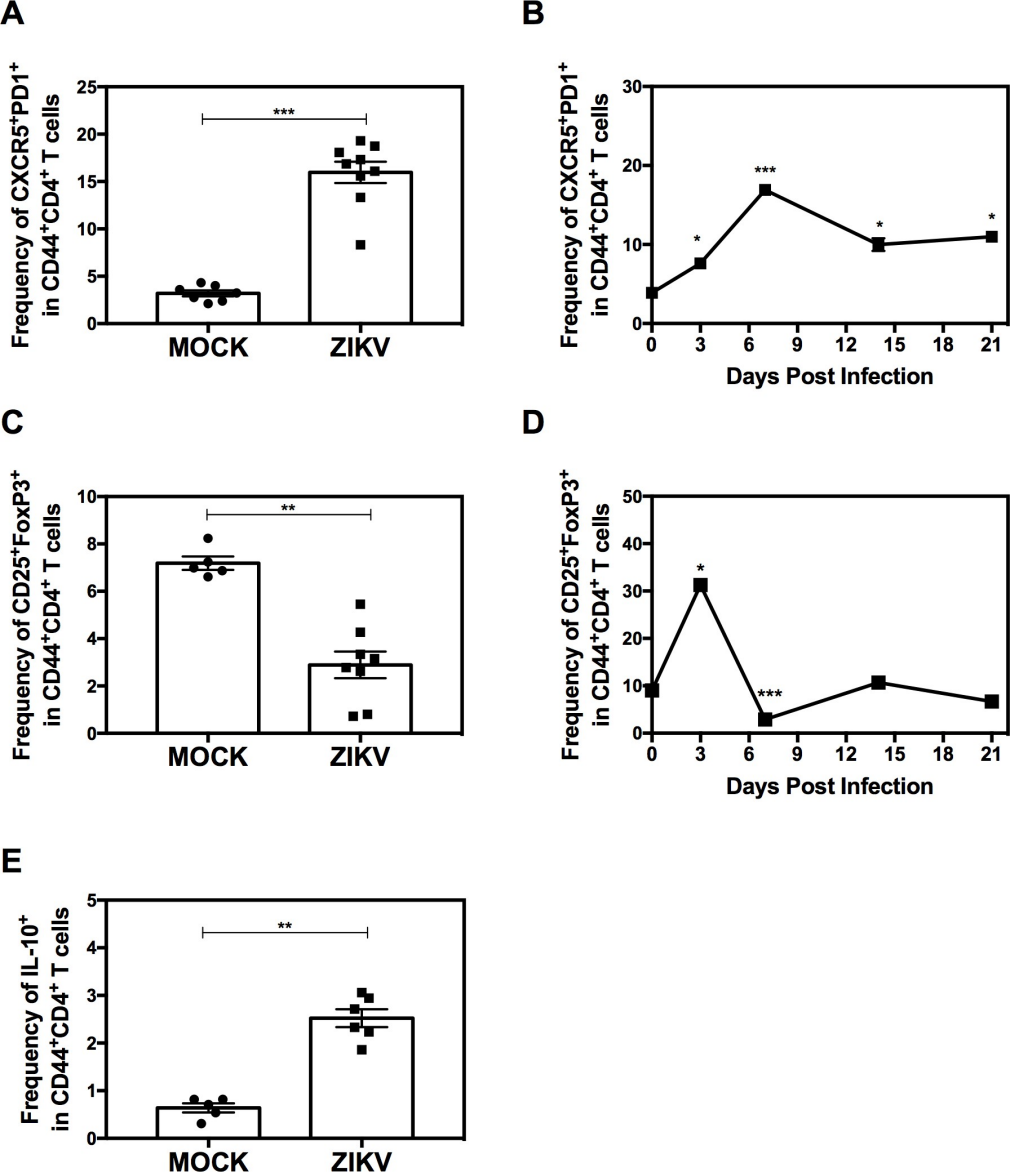
Kinetics of the follicular helper and regulatory CD4^+^ T cell responses in the *LysMCre*^+^*Ifnar1*^fl/fl^ mouse model of primary ZIKV infection. Five-week-old *LysMCre*^+^*Ifnar1*^fl/fl^ C57BL/6 mice were infected retro-orbitally with 10^4^ FFU of ZIKV strain FSS13025 or were mock-infected. (**A and C**) Splenocytes were removed on day 7 post-infection and the percentage (**A**) CXCR5^+^PD1^+^CD44^+^CD4^+^ T_FH_ cells or (**C**) CD25^+^FoxP3^+^CD44^+^CD4^+^ Treg cells was assessed by flow cytometry. (**B and D**) Splenocytes were analyzed on the indicated days post-infection for the percentage (**B**) CXCR5^+^PD1^+^CD44^+^CD4^+^ T_FH_ cells or (**D**) CD25^+^FoxP3^+^CD44^+^CD4^+^ Treg cells. (**E**) Splenocytes were removed on day 7 post-infection and analyzed for the frequency of IL-10-producing CD44^+^CD4^+^ cells. Data are the mean ± SEM of *n* = 7 (A), *n* = 4 (B), *n* = 5 (C), *n* = 4 (D), *n* = 5 (E) mock-infected and *n* = 9 (A), *n* = 4–6 (B), *n* = 8 (C), *n* = 4–6 (D), *n* = 6 (E) ZIKV-infected mice. **P < 0.01, ***P < 0.001, by the Mann–Whitney *U* test. For the kinetic analysis, each time point was compared to day 0 using the Mann–Whitney *U* test, *P < 0.05, ***P < 0.001. Data are representative of two independent experiments.

To further dissect the CD4^+^ T cell response to ZIKV, we investigated two subsets that regulate Ab and cytolytic responses. T_FH_ cells are the major cell subset that provides help to B cells and promotes antiviral Ab production [33], while Treg cells play crucial roles in limiting the immune response during infection to avoid extensive tissue damage [34]. We analyzed the CD44^+^CD4^+^ T cells for expression of the T_FH_ surface markers CXCR5 and PD-1 or the Treg markers CD25 and FoxP3 (gating strategy shown in **S2A and S2B Fig**). We observed a significant expansion of T_FH_ cells that peaked on day 7 post-infection (~5-fold increase) and remained elevated thereafter for the duration of the experiment (**Fig 2A and 2B and S2A & S2C Fig**). In contrast, we detected a significant (~2-fold) reduction in the frequency of CD44^+^CD4^+^ CD25^+^FoxP3^+^ Treg cells in day 7 splenocytes from ZIKV-infected compared with mock-infected mice (**Fig 2C and S2B & S2D Fig**). Interestingly, this reduction was preceded by a marked and transient increase in the frequency of Treg cells early in the response (day 3; **Fig 2D**). This decrease in splenic Treg cells is consistent with the findings of a recent study of ZIKV infection in wildtype mice [30] and with our earlier study in *Ifnar1*-deficient mice infected with DENV [28].

IL-10 is a key CD4^+^ T cell-secreted immunoregulatory cytokine that controls viral immunity by inhibiting proinflammatory responses and preventing tissue damage (Reviewed in [35, 36]; therefore, we also examined their frequency in ZIKV-infected mice. Unexpectedly, we saw a significant expansion of IL-10-producing CD44^+^CD4^+^ splenocytes from day 7 ZIKV-infected compared with mock-infected mice (**Fig 2E**), which contrasts with the pattern of Treg cell retraction on day 7. The expression of IFNγ by IL-10^+^CD44^+^CD4^+^ T cells indicated that a substantial proportion of the IL-10-producing cells were Th1 effector cells with a regulatory phenotype, rather than Treg cells (**S2E Fig**). Collectively, these results demonstrate that, at the peak of the T cell response, ZIKV infection induces expansion of Ab-promoting T_FH_ cells and IL-10-producing CD4^+^ T cells, but suppresses the population of Treg cells.

### Requirement for CD4^+^ T cells for induction of virus-specific IgG, but not for the CD8^+^ T cell response or viral control, after intravenous infection with ZIKV

**Fig 3:**
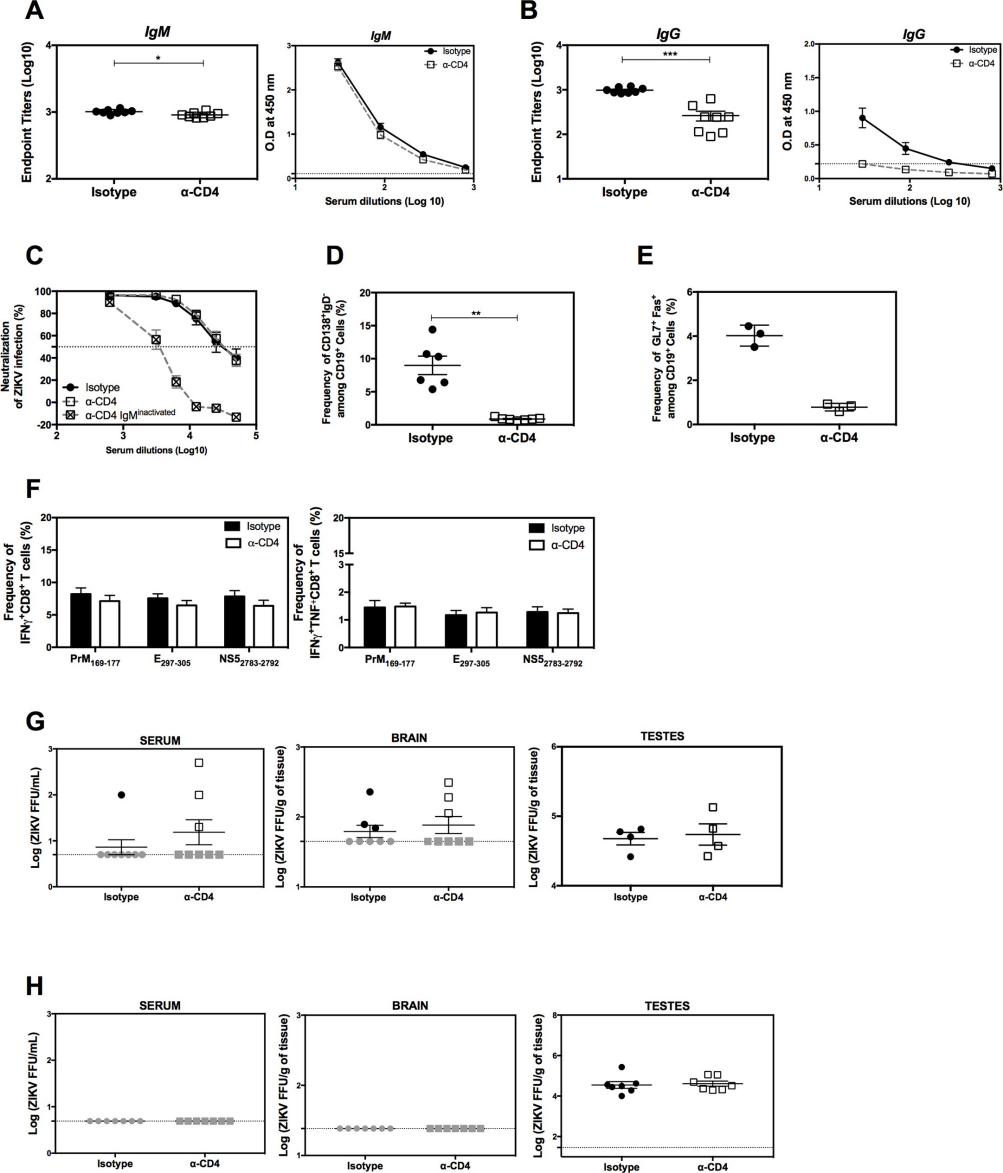
Contribution of CD4^+^ T cells to Ab and CD8^+^ T cell responses and to viral control during primary ZIKV infection in *LysMCre*^+^*Ifnar1*^fl/fl^ mice. *LysMCre*^+^*Ifnar1*^fl/fl^ C57BL/6 mice were treated with a depleting anti-CD4 Ab (*n* = 8) or isotype control Ab (*n* = 8) on days −3 and −1 prior to and every 2 days after retro-orbital infection with 10^5^ FFU of ZIKV FSS13025. (**A–C**) Sera were collected on day 7 post-infection to measure anti-ZIKV IgM (**A**) and IgG (**B**) titers by ZIKV E-specific ELISA and (**C**) ZIKV neutralizing activity using a U937 DC-SIGN cell-based assay with sera from both groups and sera from the anti-CD4 depleted mice group after inactivation of IgM. Data are the mean ± SEM. (**D and E**) Splenocytes were collected on day 7 post-infection and analyzed by flow cytometry for the percentage CD138^+^IgD^−^ plasma cells (**D**) or GL7^+^Fas^+^ germinal center B cells (**E**). Data are the mean ± SEM of *n* = 6 (D) or *n* = 3 (E) isotype control mice and *n* = 7 (D) or *n* = 3 (E) anti-CD4-treated mice. (**F**) Splenocytes were collected on day 7 post-infection, stimulated with the class I-binding ZIKV peptides PrM_169-177_, E_297-305_, or NS5_2783-2792_ and analyzed by flow cytometry for the percentage IFNγ-producing (left) or IFNγ + TNF-producing (right) CD8^+^ T cells. Data are the mean ± SEM of *n* = 8 for both isotype control and anti-CD4-treated mice. (**G**) Serum, brain, and testes were harvested on day 7 post-infection from mice treated with isotype control or depleting anti-CD4 Ab and inoculated with 10^5^ FFU of ZIKV FSS13025, and infectious ZIKV titers were determined using a focus-forming assay. Data are the mean ± SEM of *n* = 8 (serum and brain), *n* = 4 (testes) for both isotype control and anti-CD4-treated mice. (**H**) Serum, brain, and testes were harvested on day 7 post-infection from mice treated with isotype control or depleting anti-CD4 Ab and inoculated with 10^3^ FFU, and infectious ZIKV levels were measured using a focus-forming assay; *n* = 7 (serum, brain, testes) for both isotype control and anti-CD4-treated mice. *P < 0.05, **P < 0.01, ***P < 0.001 by the Mann–Whitney *U* test. Data were pooled from two and three independent experiments for high and low viral challenge dose challenge, respectively.

T_FH_ cells play important roles in generating mature B cells and supporting long-lived Ab-producing plasma cells [33]. Since ZIKV infection leads to a rapid expansion in the frequency of T_FH_ cells, we next investigated the requirement for CD4^+^ T cells in generating ZIKV-specific Abs during primary infection. *LysMCre*^*+*^*Ifnar1*^*fl/fl*^ mice were treated with isotype control or anti-CD4 Ab, and serum samples were taken on day 7 post-infection for analysis of IgM and IgG reactivity to ZIKV E protein by ELISA. As an estimate of anti-ZIKV E Ab concentrations, we determined the endpoint titer (defined as the reciprocal of the highest serum dilution to give a reading twice the cutoff absorbance). Although the ZIKV E-specific IgM titer was the same in sera from control Ab- and anti-CD4-treated mice, depletion of CD4^+^ T cells significantly reduced the ZIKV E-specific IgG endpoint titer compared with the control sera (**Fig 3A and 3B**), suggesting that CD4^+^ T cell help is required for the development of the IgG, but not IgM, response to ZIKV. Surprisingly, we also found that CD4^+^ T cells were not required for generation of ZIKV-specific neutralizing Abs (**Fig 3C**). Chemical inactivation of IgM reduced the neutralizing capacity of serum samples from anti-CD4-treated mice, indicating that this isotype was largely responsible for the neutralizing activity at day 7 after infection (**Fig 3C)**. To verify these findings, we also analyzed the Ab response at 10 days post-infection (**S3A and S3B Fig**). Although we again observed that CD4^+^ T cells were not required to generate ZIKV-specific IgM Abs, there was a marked reduction in neutralizing activity in the sera from anti-CD4 Ab-treated compared with control mice, consistent with production of IgG neutralizing Abs at this later time point during the primary infection (**S3C Fig**). These data therefore show that CD4^+^ T cells are not required for generation of the neutralizing Ab response at the peak of the T cell response at day 7 post-infection; however, they do not exclude the possible involvement of CD4^+^ T cells at later times. Indeed, analysis of splenocytes on day 7 post-infection revealed a significant reduction in the frequencies of plasma and GC B cells in CD4^+^ T cell-depleted mice compared with control mice (**Fig 3D and 3E**), supporting the potential role of CD4^+^ T cells in virus-specific IgG production at later time points after infection.

We next asked whether CD4^+^ T cells are required for the primary CD8^+^ T cell response to ZIKV infection, as we have previously demonstrated a critical role for CD8^+^ T cells in protecting against primary ZIKV infection [22]. Splenocytes from control and anti-CD4 Ab-treated mice were isolated on day 7 post-ZIKV infection, stimulated in vitro with three immunodominant class I-restricted CD8 epitopes (PrM_169-177_, E_297-305_, or NS5_2783-2792_), and analyzed for the frequencies of CD8^+^ T cells producing IFNγ alone or and IFNγ and TNF. We observed no difference in the frequency of either CD8^+^ T cell subset between splenocytes from control and CD4^+^ T cell-depleted mice (**Fig 3F**) or in the absolute number of total CD8^+^ T cells (**S3D Fig**), effector memory (CD44^high^CD62L^low^) CD8^+^ T cells (**S3E Fig**), or IFNγ- or IFNγ^+^TNF^+^-producing CD8^+^ T cells (**S3F & S3G Fig**). Thus, the primary CD8^+^ T cell response to ZIKV does not require CD4^+^ T cell help.

To investigate the impact of CD4^+^ T cell depletion on viral clearance, *LysMCre*^*+*^*Ifnar1*^*fl/fl*^ mice were treated with control or anti-CD4 Ab prior to intravenous infection with 10^5^ or 10^3^ FFU of ZIKV FSS13025. Seven days later, infectious ZIKV particles in the serum, brain, and testes were quantified using a cell-based focus-forming assay. Although the serum and brain were devoid of infectious particles in the majority of animals at this time point, ZIKV particles were detectable in the testes at levels not significantly different between the CD4^+^ T cell-sufficient and -depleted animals (**Fig 3G and 3H**).

To better mimic viral transmission via a mosquito bite in this animal model, we also evaluated the role of CD4^+^ T cells in the anti-ZIKV response of mice infected via the intrafootpad route. Here, too, we found as shown for the intravenous route that depletion of CD4^+^ T cells reduced the magnitude of the ZIKV-specific IgG, plasma cell, and GC B cell responses (**S4A–S4E Fig**) and there were no difference between control and CD4^+^ T cell-depleted mice in the CD8^+^ T cell response (**S4F and S4G Fig**) or in viral titers in serum, brain, and testes (**S4H Fig**). Overall, these results suggest that, although CD4^+^ T cells are required for the differentiation of plasma and GC B cells and for the production of virus-specific IgG, they are not involved in viral clearance during primary ZIKV infection via either the intravenous (RO) or intrafootpad routes, which contrasts with the role of CD8^+^ T cells [22].

### Contribution of memory CD4^+^ T cells to viral clearance in mice after intravenous ZIKV infection

**Fig 4:**
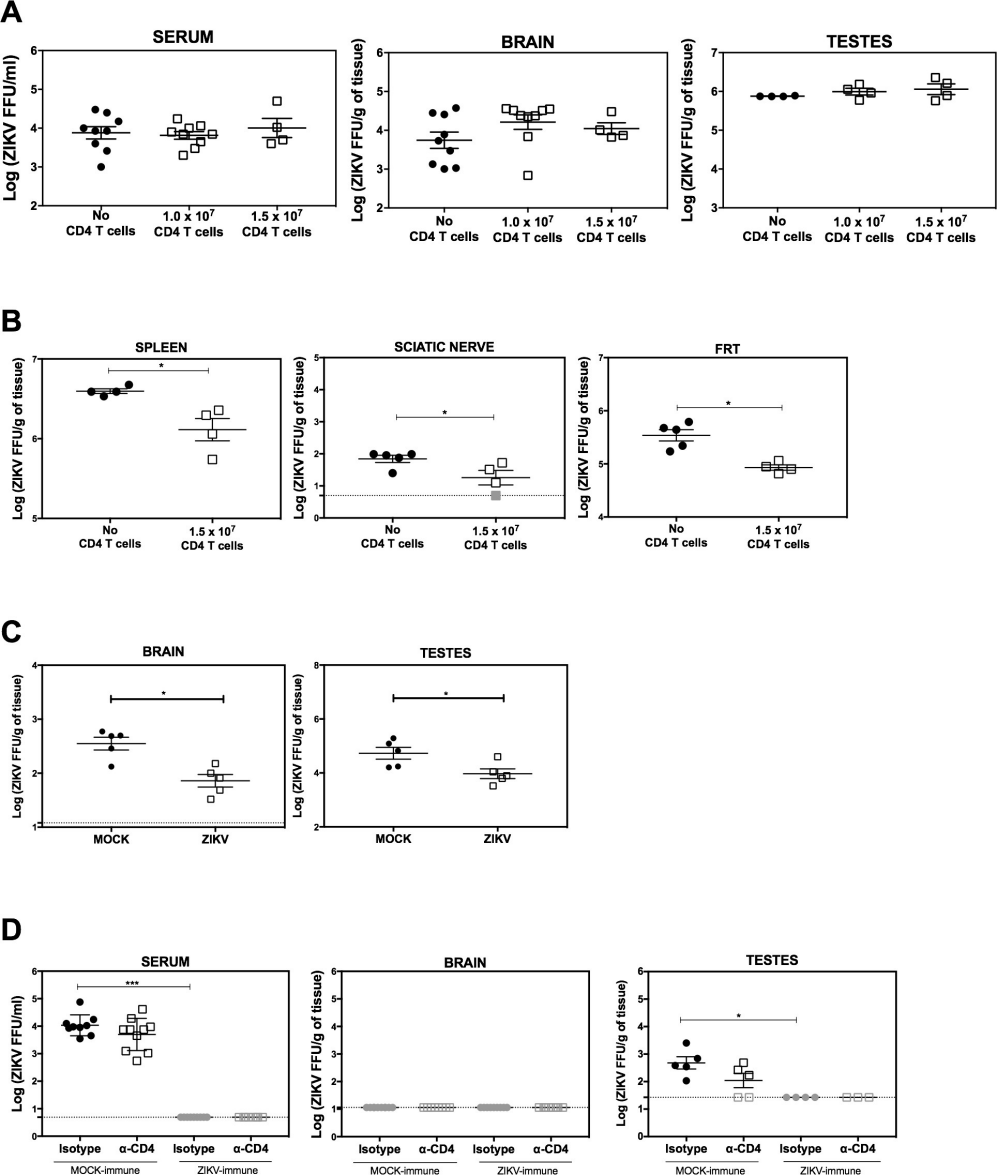
Contribution of memory CD4^+^ T cells to ZIKV clearance in *LysMCre*^+^*Ifnar1*^fl/fl^ mice. *LysMCre*^+^*Ifnar1*^fl/fl^ C57BL/6 mice were retro-orbitally infected with 10^4^ FFU of ZIKV strain FSS13025. After 30 days, CD4^+^ T cells were purified from the spleens and 10^7^ or 1.5 × 10^7^ cells were transferred into naïve *LysMCre*^+^*Ifnar1*^fl/fl^ C57BL/6 mice. One day later, mice were infected retro-orbitally with (**A**) 10^5^ FFU (*n* = 9 for serum and brain and *n* = 4 for testes) or (**B**) 10^3^ FFU of ZIKV strain FSS13025 (*n* = 4 for mice receiving CD4^+^ T cells and *n* = 5 for control mice receiving no T cells). The indicated organs were isolated 3 days later and infectious ZIKV titers were determined using a focus-forming assay. (**C**) *LysMCre*^+^*Ifnar1*^fl/fl^ C57BL/6 mice were immunized subcutaneously with a mixture of six immunodominant peptides (*n* = 5) or DMSO (*n* = 5) on days −28 and −14, and infected with 10^5^ FFU of ZIKV FSS13025 on day 0. Three days later, the brain and testes were removed and infectious ZIKV titers were determined using a focus-forming assay. (**D**) *LysMCre*^+^*Ifnar1*^fl/fl^ C57BL/6 mice were infected with 10^4^ FFU of ZIKV FSS13025 on day 0 and treated with CD4-depleting antibody or isotype control Ab on day-3 and day-1 prior to challenge with 10^3^ FFU of ZIKV FSS13025 on day 28 post-priming. Three days later, infectious ZIKV titers in serum, brain, and testes were determined using a focus-forming assay. Data are the mean ± SEM. *P < 0.05, by the Mann–Whitney *U* test.

Since our data indicate that CD4^+^ T cells do not contribute to ZIKV clearance early (day 7) after primary infection via the RO route, we asked whether memory CD4^+^ T cells, which become detectable at days 21–28 after primary infection, might be more effective. To this end, we infected mice RO with ZIKV FSS13025 and isolated splenocytes after 34 days. CD4^+^ T cells were purified and injected (10^7^ or 1.5 × 10^7^) RO into naïve *LysMCre*^*+*^*Ifnar1*^*fl/fl*^ mice, which were then inoculated RO with high or low doses of ZIKV FSS13025 (10^5^ or 10^3^ FFU, respectively). Although the transferred CD4^+^ memory T cells had no effect on infectious viral burden in the serum, brain, or testes on day 3 post-infection in the high dose-infected mice (**Fig 4A**), we observed a significant reduction in viral burden in some tissues (e.g., spleen, sciatic nerve, and female reproductive tract [FRT]) of mice injected with memory CD4^+^ T cells and inoculated with a low dose of ZIKV (**Fig 4B**). Brain and serum did not show a significant reduction in viral particles and are not represented. These data suggest that memory CD4^+^ T cells can contribute to ZIKV clearance from some, but not all, tissues during infection with low doses of ZIKV.

To explore the protective role of the anti-ZIKV CD4^+^ T cell response, we next employed a variety of additional approaches. First, *LysMCre*^*+*^*Ifnar1*^*fl/fl*^ mice were immunized with the six immunodominant Th1 ZIKV epitopes on days −28 and −14 (or mock-immunized with adjuvant alone), followed by inoculation with 10^5^ FFU of ZIKV FSS13025 on day 0. Three days later, viral titers in the brain and testes were measured. Notably, immunization with the CD4^+^ T cell epitopes resulted in a significant reduction in viral burden in both organs of ZIKV epitope-immunized mice compared with the mock-immunized mice (**Fig 4C**), confirming that memory CD4^+^ T cells could contribute to viral clearance.

Second, we asked whether memory CD4^+^ T cells contribute to viral clearance during secondary ZIKV infection. *LysMCre*^+^*Ifnar1*^fl/fl^ mice were mock- or ZIKV-inoculated RO on day 0, and then treated with a control or CD4-depleting Ab on day-3 and day-1 prior to challenge with a non-lethal dose of ZIKV on day 28 post-priming. Measurement of viral particles in the serum, brain, and testes revealed almost complete eradication of virus in ZIKV-primed mice compared with mock-primed mice on day 3 after secondary ZIKV infection, with no apparent difference between anti-CD4-treated and control Ab-treated mice (**Fig 4D**). The anti-CD4-treated mice showed reduced frequencies of plasma cells and GC B cells compared with control mice (**S5A and S5B Fig**), consistent with the role of CD4^+^ T cells in production of memory anti-ZIKV Ab responses. In contrast, the number of memory CD8^+^ T cells producing IFNγ^+^TNF^+^ was higher in CD4-depleted compared with control mice (**S5C and S5D Fig)**. Analysis of CD4^+^ T cell subsets revealed an increase in T_FH_ cells and IFNγ-, IFNγ^+^ TNF^+^-, and IL-2-producing CD4^+^ T cells and a decrease in Treg cells in Mock-immune compared with ZIKV-immune mice (**S5E–S5G Fig)**, which is consistent with the observation that Treg cell number peaks at day 3 after primary infection (**Fig 2D**). These data thus reveal the presence of a memory CD4^+^ T cell response in ZIKV-immune mice, but indicate that this memory CD4^+^ T cell response is not required to control viral clearance early after a secondary ZIKV challenge.

Third, we examined whether CD4^+^ T cells could contribute to protection against ZIKV infection in fully deficient *Ifnar1*^−/−^ mice, which are more susceptible than *LysMCre*^+^*Ifnar1*^fl/fl^ mice to ZIKV infection. *Ifnar1*^−/−^ mice were immunized with a mixture of the ZIKV peptides, boosted two weeks post-immunization, and then challenged 14 days later with a lethal dose of ZIKV. Body weight, clinical signs of disease, and mortality were monitored daily for 15 days post-challenge. Although fewer peptide-immunized mice succumbed to ZIKV infection compared with mock-immunized mice (~50% and 75%, respectively), both groups of mice showed similar clinical disease scores and no significance difference was observed between the two groups (**S6A–S6D Fig)**. To assess a possible role for CD4^+^ T cells in protection, *Ifnar1*^−/−^ mice were injected with a CD4^+^ T cell-depleting Ab or isotype control Ab on days −3 and −1 prior to lethal dose of ZIKV. We daily monitored the infected mice and detected no significant difference in weight loss, clinical disease score, or mortality between mice treated with anti-CD4 Ab or isotype control Ab (**S7A–S7D Fig)**. Both groups succumbed to ZIKV infection after nine days of infection (**S7A–S7D Fig)**. Similar results were obtained even upon secondary challenge of ZIKV-primed *Ifnar1*^−/−^ mice (**S7E and S7F Fig**). Specifically, *Ifnar1*^−/−^ mice were injected with a CD4^+^ T cell-depleting Ab or isotype control Ab on days −3 and −1 prior to priming with a sub-lethal dose of ZIKV, and then every week thereafter for 4 weeks. On day 30, the mice were challenged with a lethal dose of ZIKV. We detected no significant difference in weight loss, clinical disease score, or mortality between mice primed with ZIKV and treated with anti-CD4 Ab or isotype control Ab for 30 days prior to secondary ZIKV infection (**S7E and S7F Fig**). Thus, CD4^+^ T cells do not appear to play an essential role in protecting against lethal primary or secondary infection with ZIKV.

Taken together, the results of the adoptive cell transfer and peptide immunization experiments using *LysMCre*^+^*Ifnar1*^fl/fl^ mice indicate that CD4^+^ T cells do play a role in controlling viral burden in ZIKV-infected mice under certain conditions, such as low-dose secondary infection and peptide vaccination. Additionally, experiments using *LysMCre*^+^*Ifnar1*^fl/fl^ and *Ifnar1*^−/−^ mice show that CD4^+^ T cells also play a role in Ab production during secondary infection but are not required to protect against systemic lethal challenge.

### Vigorous CD4^+^ T cell response after primary intravaginal ZIKV infection in *LysMCre*^+^*Ifnar1*^fl/fl^ mice

**Fig 5:**
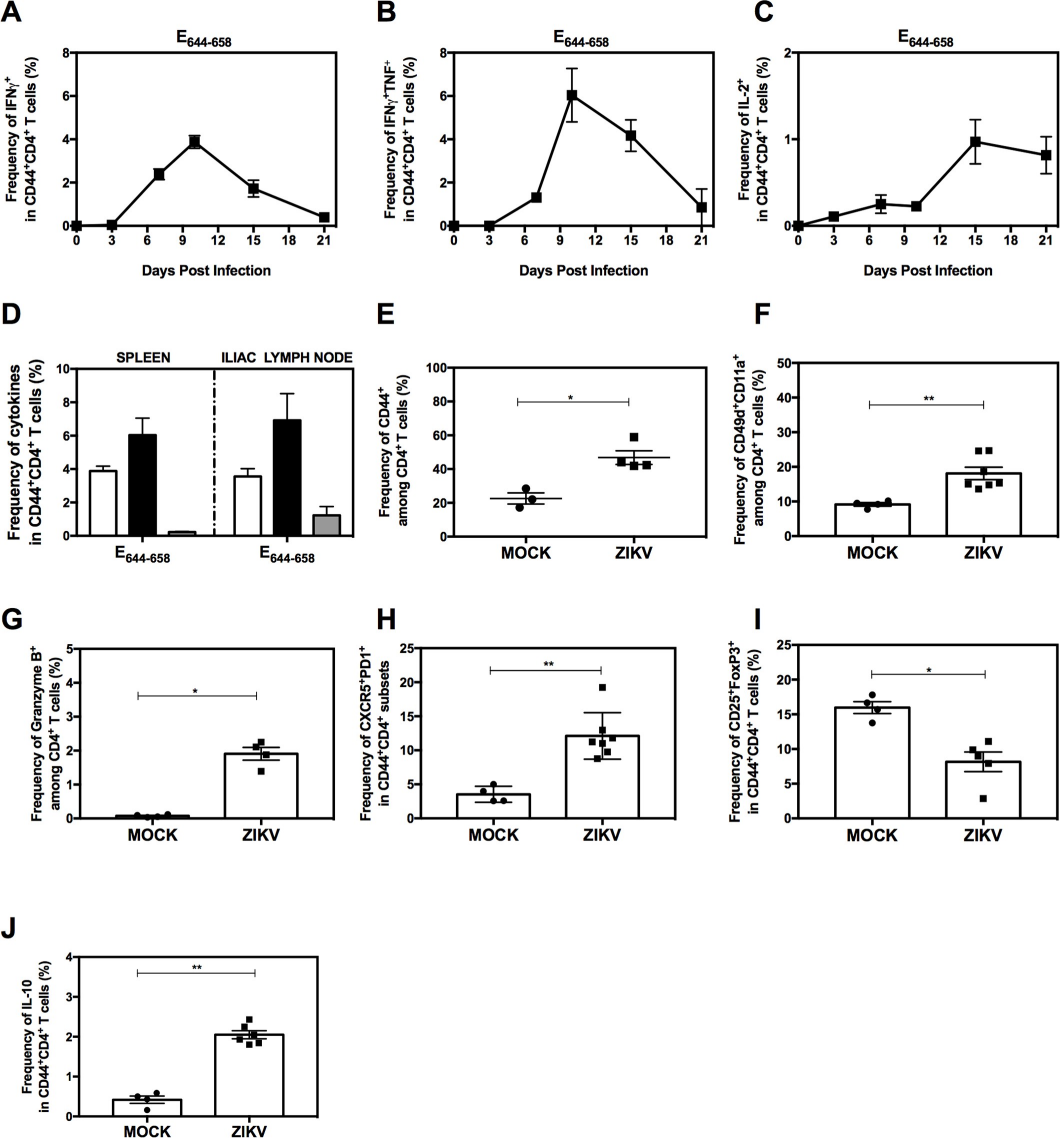
Characterization of the CD4^+^ T cell response after primary intravaginal ZIKV infection in *LysMCre*^+^*Ifnar1*^fl/fl^ mice. *LysMCre*^+^*Ifnar1*^fl/fl^ C57BL/6 female mice (8- to 9-week-old) were treated with progesterone and infected via IVag route with 10^5^ FFU of ZIKV FSS13025 3 days later. (**A–C**) On the indicated days, splenocytes (*n* = 4–8 mice) were stimulated *in vitro* with E_644-658_ FSS13025 peptide and analyzed by flow cytometry for the frequency of CD44^+^CD4^+^ T cells producing (**A**) IFNγ, (**B**) IFNγ + TNF, and (**C**) IL-2. (**D**) As described for (A–C) except cells were harvested from the spleen and iliac lymph nodes (*n* = 8) on day 10 post-infection, stimulated *in vitro*, and analyzed for the frequency of CD44^+^CD4^+^ T cells producing IFNγ (white bars), IFNγ + TNF (black bars), or IL-2 (gray bars). (**E–J**) On day 10 post-infection, splenocytes were analyzed for the frequency of (**E**) CD44^+^CD4^+^ T cells, (**F**) CD49d^+^CD11a^+^ cells, and (**G**) granzyme B^+^ cells, (**H**) T_FH_ (CXCR5^+^PD1^+^CD44^+^CD4^+^) cells, (**I**) Treg cells (FoxP3^+^CD25^+^CD4^+^CD44^+^) cells, and (**J**) IL-10-producing CD44^+^CD4^+^ T cells. All experiments were performed twice. Data are the mean ± SEM of *n* = 3 (E), *n* = 4 (F), *n* = 4 (G), *n* = 4 (H), *n* = 4 (I), *n* = 4 (J) mock-infected and *n* = 4 (E), *n* = 7 (F), *n* = 4 (G), *n* = 7 (H), *n* = 5 (I), *n* = 6 (J) ZIKV-infected mice. *P < 0.05, **P < 0.01 by the Mann–Whitney *U* test.

Given the increasing evidence that ZIKV can be transmitted via sexual contact [1], we were also interested in determining whether a protective CD4^+^ T cell response is activated via this route of infection. We previously described a model of sexual transmission of ZIKV using *LysMCre*^+^*Ifnar1*^fl/fl^ mice [5]. In this model, hormonal changes were found to influence ZIKV transmission, since mice were more susceptible to intravaginal (IVag) ZIKV infection and the virus persisted in the female reproductive tract (FRT) for longer periods during the diestrus-like phase relative to estrus-like phase [5]. Therefore, for the experiments performed here, we induced a diestrus-like phase by treating *LysMCre*^+^*Ifnar1*^fl/fl^ mice with progesterone at day −3 prior to intravaginal infection with a high dose (10^5^ FFU) of ZIKV FSS13025. Mice were confirmed to be in a diestrus-like phase by vaginal cytology prior to inoculation [37]. Splenocytes were prepared on days 3, 7, 10, 15, and 21 after infection, stimulated *in vitro* with the immunodominant epitope E_644-658_, and analyzed by flow cytometry. As shown in **Fig 5**, an increase in the frequency of IFNγ- and IFNγ + TNF-producing CD44^+^CD4^+^ T cells was detectable on day 6, peaked at day 10, and waned thereafter. In contrast, IL-2-producing CD44^+^CD4^+^ T cells were already detectable on day 3 but were most abundant on day 15 post-infection (**Fig 5A–5C**). To investigate the localized immune response to IVag ZIKV infection, we analyzed CD4^+^ T cell activation in the local (iliac) draining lymph nodes (LNs) on day 10 after infection. Despite the much lower absolute number of cells compared with the spleen, we detected a similar percentage of IFNγ-, IFNγ + TNF-, and IL-2-expressing CD44^+^CD4^+^ T cells in the iliac LNs and the spleen (**Fig 5D**). Notably, we also observed the same pattern of expansion of CD4^+^ T cell subsets in the spleen on day 10 after IVag ZIKV infection as we had on day 7 after RO infection. Thus, the frequencies of activated (CD44^+^), antigen-experienced (CD49d^+^CD11a^+^), cytolytic (granzyme B^+^), and T_FH_ CD4^+^ T cells in the spleen were all increased (**Fig 5E–5H**), whereas the Treg cell frequency was reduced (**Fig 5I**). Moreover, we also observed an increase in IL-10-producing CD4^+^ T cells (**Fig 5J**). CD4^+^ T cells in the iliac LNs of intravaginally infected mice showed an identical pattern of expansion of T_FH_ and IL-10-producing CD4^+^ T cells and reduction in Treg cells on day 10 post-infection (**S8A–S8C Fig**). Thus, IVag ZIKV infection induced a vigorous Th1 CD4^+^ T cell response in the spleen and draining LNs comparable to that observed in the spleen after RO infection.

Since the T cell response peaked later after IVag ZIKV infection than after RO infection, we examined the frequency of CD8^+^ effector memory (CD44^high^CD62L^−^) and CD8^+^ central memory (CD44^high^CD62L^+^) T cell subsets on day 10 after IVag infection with ZIKV FSS13025. We found a significant expansion (~2.5-fold) of effector memory CD8^+^ T cells and antigen-experienced (CD11a^high^) CD8^+^ T cells (~6-fold), and a concomitant reduction (~2.5-fold) in central memory CD8^+^ T cells in the ZIKV-infected compared with mock-infected mice on day 10 post-infection (**S9A & S9B Fig**). In addition, splenic CD8^+^ T cells producing IFNγ and IFNγ + TNF were present after *in vitro* stimulation with the class I-restricted ZIKV E_297-305_ epitope (**S9C Fig**). Taken together, these results indicate that IVag infection with ZIKV promotes vigorous systemic CD4^+^ and CD8^+^ T cell responses.

### CD4^+^ T cell-mediated regulation of the antiviral Ab response, control of local viral burden, and protection from lethality following intravaginal infection with ZIKV

**Fig 6:**
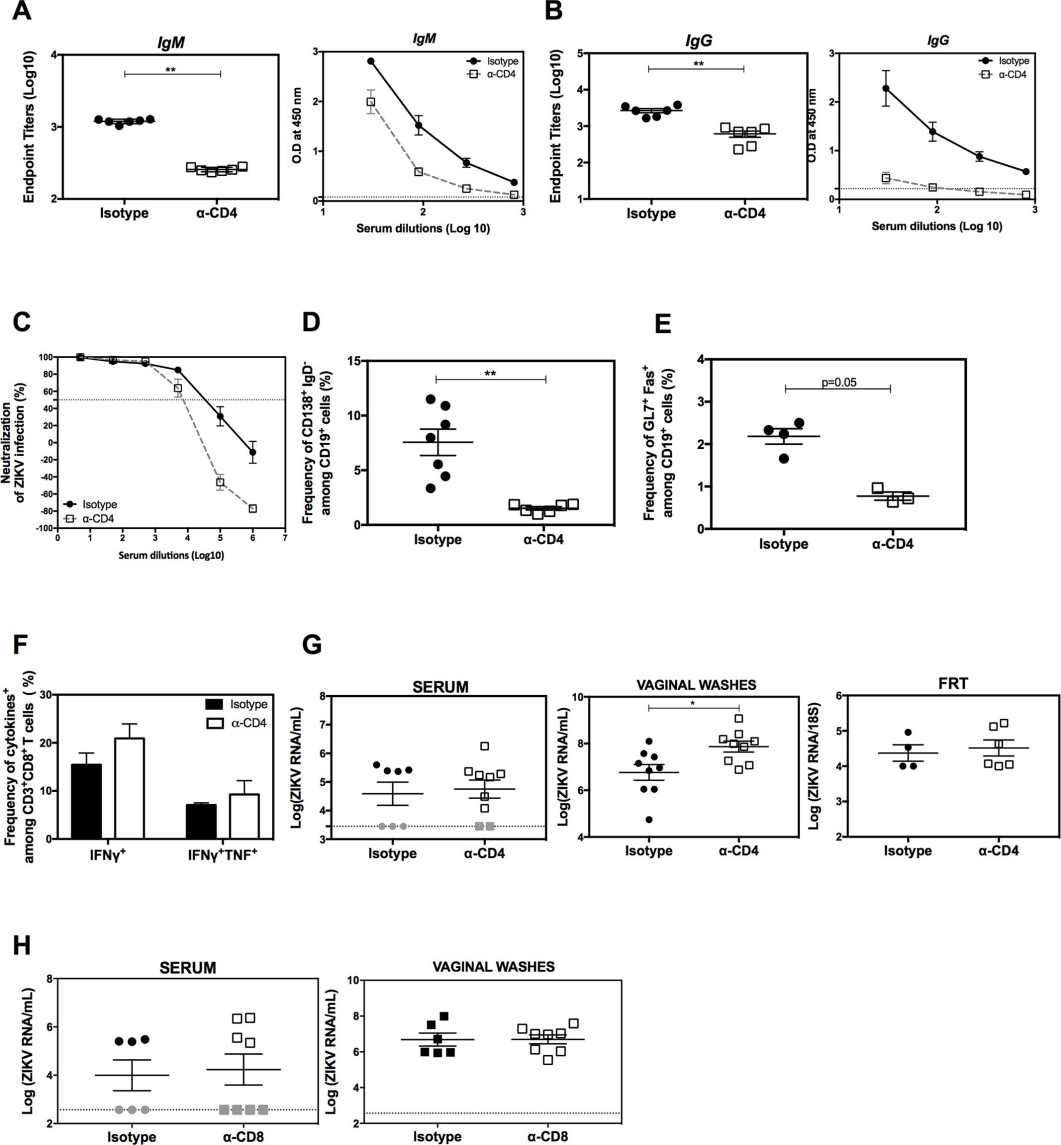
Contribution of CD4^+^ T cells to Ab production, CD8^+^ T cell response, and local viral control during primary intravaginal ZIKV infection of *LysMCre*^+^*Ifnar1*^fl/fl^ mice. Female *LysMCre*^+^*Ifnar1*^fl/fl^ C57BL/6 mice were treated with a depleting anti-CD4 Ab (*n* = 7) or isotype control Ab (*n* = 6) on days −3 and −1 prior to intravaginal infection with 10^5^ FFU of ZIKV FSS13025. Mice were also treated with progesterone on day −3. (**A–C**) Sera were collected on day 10 post-infection to measure anti-ZIKV IgM (A) and IgG (B) titers by ZIKV E-specific ELISA and neutralizing activity (C) using a U937 DC-SIGN cell-based flow cytometric assay. (**D and E**) Splenocytes were collected on day 10 post-infection and analyzed by flow cytometry for the percentage plasma cells (CD138^+^IgD^−^) (D) or germinal center B cells (GL7^+^Fas^+^) (E). Data are the mean± SEM of *n* = 7 (D) or 4 (E) for isotype control mice and *n* = 6 (D) or 3 (E) for anti-CD4-treated mice. (**F**) Splenocytes were collected on day 10 post-infection, stimulated with the immunodominant CD8^+^ T cell ZIKV epitope E_297-305_, and analyzed by flow cytometry for the percentage IFNγ- or IFNγ + TNF-producing CD8^+^ T cells. Data are the mean ± SEM of *n* = 8 mice/group. (G) Infectious ZIKV particles were measured in serum (*n* = 7, *n* = 9), vaginal washes (*n* = 9, *n* = 9), and the female reproductive tract (FRT) (*n* = 4, *n* = 6) on day 10 post-infection for the isotype control or anti-CD4 treated groups. (H) As described for (G) except mice were treated with an anti-CD8 Ab (*n* = 6) or isotype control Ab (*n* = 8) before infection, only serum and vaginal washes are represented. *P < 0.05, **P < 0.01 by the Mann–Whitney *U* test.

Our results thus far reveal a mixed role for CD4^+^ T cells in promoting Ab and viral clearance to ZIKV infection via the RO route. To determine whether this was also the case for the primary response to IVag infection, we performed a similar analysis using progesterone-pretreated *LysMCre*^+^*Ifnar1*^fl/fl^ mice that were infused with a control or depleting anti-CD4 Ab before IVag ZIKV infection. Analysis of serum on day 10 post-infection revealed that, in contrast to the findings with mice infected RO, depletion of CD4^+^ T cells significantly reduced the titers of ZIKV-specific IgM and IgG as well as the anti-ZIKV neutralizing Ab activity compared with control mice (**Fig 6A–6C**). We observed a reduction in the frequency of splenic plasma cells and GC B cells (**Fig 6D and 6E**) in CD4-depleted mice but saw no effect on the CD8^+^ T cell response, as reflected by the frequency of IFNγ- and IFNγ+ TNF-producing cells after *in vitro* stimulation of splenocytes with E_297-305_ ZIKV epitope (**Fig 6F**), on day 7 post-infection.

To assess the requirement for CD4^+^ T cells for viral clearance after IVag infection, we quantified infectious ZIKV particles in serum, vaginal washes, and the FRT on day 10 post-IVag infection of control or anti-CD4 Ab-treated *LysMCre*^+^*Ifnar1*^fl/fl^ mice. Although the viral burden in serum and FRT was unaffected by CD4^+^ T cell depletion, ZIKV RNA levels were significantly higher in the vaginal washes of anti-CD4-treated compared with isotype control Ab-treated mice (**Fig 6G**). To determine whether viral clearance from vaginal washes was mediated by CD4^+^ T cells alone, CD8^+^ T cells were depleted by injection of anti-CD8 Ab on days −3 and −1 before IVag infection with ZIKV, and serum and vaginal washes were analyzed for ZIKV RNA levels at 10 days post-infection. We detected no difference in viral RNA levels between anti-CD8 Ab and isotype control Ab-treated mice in either sample (**Fig 6H**), indicating that CD8^+^ T cells are likely not involved in control of viral burden following IVag infection. However, animals lacking CD8^+^ T cells showed an enhanced CD4^+^ T cell response, with increased IFNγ- and IFNγ+ TNF-producing cell frequencies and numbers in the spleen (**S10A & S10B Fig**) and increased frequencies in the iliac lymph nodes (**S10C Fig**), suggesting that the CD4^+^ T cell response to ZIKV compensates for the absence of CD8^+^ T cells by increasing the abundance of Th1 cells. To confirm and extend this result showing CD4^+^ T cell contribution to protection against IVag ZIKV infection in *LysMCre*^+^*Ifnar1*^fl/fl^ mice, we depleted CD4^+^ T cells in *Ifnar1*^−/−^ mice on days −3 and −1 before IVag infection with a lethal dose of ZIKV. Only 22% of CD4-depleted mice survived to day 15 post-infection compared with 78% of control Ab-treated mice (**S11A Fig),** and the CD4^+^ T cell-depleted animals showed greater body weight loss and more severe clinical disease scores than the control animals (**S11B–S11D Fig)**. Collectively, the results of the experiments with *LysMCre*^+^*Ifnar1*^fl/fl^ and *Ifnar1*^−/−^ mice infected via the IVag route indicate that CD4^+^ T cells are required to mount an efficient Ab response, to control the local viral burden, and to reduce clinical signs and mortality following IVag infection.

## Discussion

The role of CD4^+^ T cells in the regulation of anti-ZIKV adaptive immunity has yet to be defined. In this study, we explored the CD4^+^ T cell response to primary RO and IVag infection with ZIKV using *LysMCre*^+^*Ifnar1*^fl/fl^ mice, in which the type I IFN receptor is absent from myeloid cells but present on T and B cells. We previously used these mice to study the CD8^+^ T cell response to RO ZIKV infection [22] and to establish a model of sexually transmitted ZIKV infection [5]. In the present study, we demonstrate that RO and IVag ZIKV infection both induce robust antigen-specific Th1, T_FH,_ plasma cell, GC B cell, IgM, and IgG responses, and that CD4^+^ T cells contribute to the generation of Ab responses, but not CD8^+^ T cell responses, and to the control of viral infection. Thus, provision of help for Ab responses may be a dominant feature of the protective role of CD4^+^ T cells during primary ZIKV infection.

Using the *LysMCre*^+^*Ifnar1*^fl/fl^ C57BL/6 mouse model, we first mapped the CD4^+^ T cell response to ZIKV proteins and identified immunodominant epitopes from structural and nonstructural proteins. Similar to our findings here, previous work has shown that the human CD4^+^ T cell response to ZIKV is targeted against epitopes in both structural and nonstructural proteins, with the most immunodominant epitopes being located in ZIKV E, NS1, and NS5 [38–40]. In our study, the strongest response following ZIKV infection via both RO and IVag routes was to the E_644-658_ epitope, which is located in domain III (EDIII) of the ZIKV E protein. EDIII is exposed on the virion surface and is one of the main targets of neutralizing Ab responses to flaviviruses [41–43]. Indeed, several groups have identified highly neutralizing ZIKV EDIII-specific Abs [12, 17, 44, 45]. The fact that the most immunodominant ZIKV epitope for CD4^+^ T cells is in EDIII could suggest that robust T cell help drives the production of EDIII-reactive and neutralizing Abs.

Although recent work has shown that ZIKV infection induces activation and expansion of CD4^+^ T cells with a Th1 phenotype [30, 31], those studies did not analyze the antigen specificity of the response. Here, we confirmed the earlier findings that Th1 CD4^+^ T cells with cytotoxic activity are the major cell type elicited during the primary ZIKV response. We also found that the percentage and absolute number of Treg cells were both reduced at the peak of the T cell response to ZIKV infection via RO and IVag routes. A similar effect on Treg cells was observed in mouse models of systemic ZIKV [30] and DENV [28] infection. We also detected an expansion of Treg cells at an early time point, day 3, after ZIKV infection. Winkler and colleagues examined the kinetics of the CD4^+^FoxP3^+^ cell response in C57BL/6 mice infected with ZIKV intraperitoneally [31]; however, these authors detected a reduction, not an expansion, of CD4^+^FoxP3^+^ cells on day 3. This apparent discrepancy may have been due to a difference in analytical gating strategy. Whereas we analyzed the CD4^+^CD44^+^FoxP3^+^CD25^+^ T cell subset, Winkler et al. examined the larger pool of CD4^+^FoxP3^+^ cells, which may have limited their capacity to detect subtle changes in minor subpopulations [31]. We demonstrated that ZIKV infection via the RO and IVag routes generates IL-10-producing Th1 cells, which also possess regulatory activity but are distinct from the CD25^+^FoxP3^+^ subset that develops in the thymus [35]. In future, studies examining the relationship between these T cell subsets in regulating the balance between promoting antiviral immunity and restraining inflammatory processes during ZIKV infection will be critical for deciphering the mechanisms of adaptive immune protection against ZIKV.

Our study demonstrates that RO or IVag infection with ZIKV induces T_FH_ cells, which are required for GC development and function [33, 46]. The finding that CD4^+^ T cells are necessary for the generation of plasma and GC B cell responses, in addition to the production of ZIKV-specific IgG after infection via either the RO, intrafootpad or IVag routes, suggests that T_FH_ cells could control B cell maturation and Ab production in response to ZIKV infection. Depletion of CD4^+^ T cells impaired the generation of neutralizing Abs during the peak of the T cell response following IVag, but not intravenous, ZIKV infection, which is probably due to the different times at which the T cell response peaks post-infection via the two routes. Similarly, production of ZIKV-specific IgM was disrupted in the absence of CD4^+^ T cells mainly after IVag but not RO infection. Moreover, the neutralizing Ab response at the peak of the T cell response to RO infection was mainly due to IgM, which is consistent with the response to WNV [47]. These observations suggest that the T_FH_ and, possibly, Th1 responses (which can regulate the magnitude and quality of T_FH_ responses [48, 49]) differentially regulate the anti-ZIKV Ab response during systemic versus mucosal infection.

Scott and colleagues recently reported the effects of prior cellular and humoral immunity on subsequent IVag ZIKV exposure in mice [50]. In particular, they showed that CD4^+^ T cells were required for the production of ZIKV-specific IgG, but not for viral clearance during either primary or secondary infection [50]. Similarly, another study observed that CD4^+^ T cells were not necessary to control secondary subcutaneous ZIKV infection of mice [51]. In our study, we showed using peptide vaccination and adoptive cell transfer approaches that memory CD4^+^ T cells contributed to viral clearance from multiple tissues during systemic challenge, but they were not required to protect against lethal challenge. Two potential explanations for the discrepancies between these studies and ours are the use of different mouse models (strain and age) and different ZIKV challenge doses. However, consistent with the finding by Scott and colleagues [50], we found that CD4^+^ T cells were required for generation of plasma cell, GC B cell, and Ab responses upon infection via both systemic and mucosal routes, suggesting that the anti-ZIKV CD4^+^ T cell response plays a dominant role in driving Ab production, irrespective of the infection route.

In our study, we used peptide vaccination and adoptive transfer experiments to demonstrate that ZIKV-specific memory CD4^+^ T cells can promote viral clearance during RO ZIKV infection. However, this was only true for adoptive transfer before challenge with a low dose of virus. Based on our previous study demonstrating a critical role for CD8^+^ T cells in controlling RO ZIKV infection [22], we speculate that high-dose viral challenge more effectively activates multiple innate immune and CD8^+^ T cell responses than does low-dose challenge. In the present study, CD4^+^ T cells play a dominant role in promoting humoral immunity to ZIKV after infection via both systemic and genital mucosal routes, and CD4^+^ T cells, but not CD8^+^ T cells, contribute to local control of ZIKV infection in the vagina and protect against lethal disease following IVag ZIKV challenge. Thus, the viral challenge dose and the route of exposure may differentially dictate the quantity and quality of induced T_FH_ cells.

In conclusion, our data provide evidence for qualitatively different protective roles of CD4^+^ T cells in ZIKV infection via the RO and IVag routes. Since we previously reported that CD4^+^ T cells play a limited role in protecting pregnant mice against primary infection via the RO route [26], our results here highlight the importance of exploring whether CD4^+^ T cells are protective when infection occurs intravaginally during pregnancy. The majority of current work on ZIKV vaccines is focused on eliciting a neutralizing Ab response. However, based on our data suggesting that T_FH_ and antigen-specific Th1 cells may regulate the magnitude and quality of the anti-ZIKV Ab response, it may be prudent to design ZIKV vaccines that induce robust CD4^+^ T cell responses in addition to Abs. This study provides the foundation for further dissection of the H-2^b^-restricted CD4^+^ T cell response to ZIKV in various mouse models, including pregnant mice infected via systemic and mucosal routes in both natural infection and vaccination contexts. Such studies should help to identify the precise features of the anti-ZIKV CD4^+^ T cell response that can be manipulated to generate ZIKV vaccines that are safe and effective against infection in multiple contexts, including pregnancy and sexual transmission.

## Methods

### Ethics statement

All experiments were performed in strict accordance with recommendations set forth in the National Institutes of Health Guide for the Care and Use of Laboratory Animals and approved by the Institutional Animal Care and Use Committee at the La Jolla Institute for Allergy and Immunology (protocol number APO28-SS1-0615 and AP00001029). Both ZIKV strains (MR766 and FSS13025) were obtained from the World Reference Center for Emerging Viruses and Arboviruses with Institutional Review Board approval. All samples were anonymized.

### Mouse experiments

*LysMCre*^*+*^*Ifnar1*^*fl/fl*^ *and Ifnar1*^*−/−*^ mice were bred under pathogen-free conditions at La Jolla Institute for Allergy & Immunology. Sample sizes were based on similar studies [5, 22]. None of the experiments were randomized or blinded.

### Virus culture and titration

Asian lineage strain FSS13025 was isolated from a 3-year-old boy in Cambodia in 2010 [52], and African lineage strain MR766 was isolated from a sentinel rhesus monkey in Uganda in 1947 [53]. Both viruses were cultured in C6/36 *Aedes albopictus* mosquito cells (American Type Culture Collection [ATCC]; Manassas, Virginia). Viral supernatants were collected 7–10 days after infection, clarified by centrifugation, and concentrated by ultracentrifugation. Viral titers were measured using a baby hamster kidney (BHK)-21 (ATCC) cell-based focus-forming assay (FFA). In brief, BHK-21 cells were plated at 2 × 10^5^ cells per well in a 24-well plate and incubated overnight in complete MEM-α medium (containing 10% fetal bovine serum [FBS], 1% penicillin/ streptomycin, and 1% HEPES) at 37°C in a 5% CO_2_ atmosphere. Cells were infected with serial dilutions of virus for 1.5 h with gentle shaking every 15 min. The medium was then aspirated and replaced with fresh complete MEM-α medium supplemented with 1% carboxymethyl cellulose (Sigma), and the cells were cultured for 2 days. Cells were then fixed with 4% formalin (Fisher), permeabilized with 1% Triton X-100 (Sigma) and blocked by addition of 10% FBS in phosphate-buffered saline (PBS). ZIKV was detected by incubation of cells with 4G2, a pan-flavivirus E protein-specific monoclonal Ab (BioXcell) for 1.5 h. Cells were washed and incubated for 1.5 h with horseradish peroxidase (HRP)-conjugated goat anti-mouse IgG (Sigma). Finally, foci were detected by incubation with True Blue substrate (KPL) and counted manually. Viral titers were expressed as log FFU/g tissue or log FFU/ml serum. Next-generation sequencing of viral stocks confirmed the absence of competing pathogens.

### Peptide prediction and synthesis

ZIKV MR766 and FSS13025 sequences were obtained from the NCBI protein database. MHC class II peptide binding affinity predictions were performed using the Immune Epitope Database (www.iedb.org) website tools using the “IEDB-recommended” method selection, as previously described [54]. Predicted binding affinities were obtained for all non-redundant 15-mer peptides that bound H-2 I-A^b^, and the peptide list was sorted by increasing consensus percentile rank and restricted to the top 1%. Peptides were synthesized by Synthetic Biomolecules as crude material (1 mg scale) and validated by mass spectrometry. Peptides for *in vitro* stimulation followed by flow cytometric analyses were synthesized and purified by reverse-phase high performance liquid chromatography to ≥95% purity. Peptides were dissolved in DMSO for use.

### Mouse infection

*LysMCre*^+^*Ifnar1*^fl/fl^ and *Ifnar1*^−/−^ mice (5- to 6-week-old males and females for RO and intrafootpad infection; 8- to 9-week-old females for IVag infection) were infected with ZIKV FSS13025 or MR766 at 10^1^, 10^2^, 10^3^, 10^4^, or 10^5^ FFU RO, 10^5^ or 10^6^ FFU IVag, and 10^5^ FFU via intrafootpad route in 10% FBS/PBS. Three days prior to IVag infection, mice were injected subcutaneously with 2 mg of progesterone (Millipore Sigma) in 100 μl of 5% ethanol, 5% Kolliphor, and 90% H_2_O to induce a diestrus-like phase, which was confirmed as previously described [5].

### CD8^+^ and CD4^+^ T cell depletion

T cell depletion Abs (CD8^+^ T cell-depleting clone 2.43 or CD4^+^ T cell-depleting clone GK1.5) were purchased from BioXCell and administered on days −3 and −1 pre-infection and every 2 days post-infection for the duration of the experiment. For long-term (30 day) depletion, mice were treated on days −3 and −1 and weekly thereafter until the end of the experiment. A rat IgG2 isotype control Ab (clone LTF-2) served as the control and was administered on days −3 and −1 pre-infection only.

### Adoptive T cell transfer

CD4^+^ T cells were isolated from donor mice 34 days after infection with 10^4^ FFU of ZIKV FSS13025. Spleens were harvested, and CD4^+^ T cells were positively selected using a CD4 T Cell Isolation Kit (Miltenyi Biotec). A total of 1 × 10^7^ or 1.5 × 10^7^ purified CD4^+^ T cells were then injected RO into the recipient mice 1 day prior to ZIKV infection. CD4^+^ T cells purified from naïve *LysMCre*^*+*^*Ifnar1*^*fl/fl*^ mouse spleens were harvested and injected into recipient mice as controls.

### Tissue collection

For preparation of serum samples, mice were sacrificed by CO_2_ inhalation and blood was collected by cardiac puncture. For vaginal washes, the vaginal canal was rinsed 3–5 times with 40 μl PBS and the washes were combined. Mice were then perfused with PBS and the desired organs were collected. For tissue FFA, organs were transferred to pre-weighed tubes containing complete MEM-α medium and a metal bead, and the tubes were then stored at −80°C until analyzed. For qRT-PCR, organs were stored in RNAlater (Invitrogen) at 4°C until analyzed.

### Quantification of virus in tissues

For the FFA, pre-weighed tubes containing frozen organs were thawed, homogenized (Tissuelyser II; Qiagen), and centrifuged. The supernatant was serially diluted, added to BHK-21 cells, and the titers were determined as described above. Titers were expressed as the log FFU/g of tissue. For qRT-PCR, RNA was isolated from tissues using an RNeasy Mini Kit (Qiagen) or from serum or vaginal washes using a Viral RNA Isolation Kit (Qiagen). qRT-PCR was performed using a qScript One-Step qRT-PCR Kit (Quanta, Bioscience) with a CFX96 Touch™ real-time PCR detection system (Bio-Rad CFX Manager 3.1). ZIKV-specific primers have been previously described [55]. Cycling conditions were: 45°C for 15 min, 95°C for 15 min, followed by 50 cycles of 95°C for 15 s and 60°C for 15 s, and a final extension of 72°C for 30 min. Viral RNA concentration was calculated using a standard curve composed of five 100-fold serial dilutions of *in vitro*-transcribed RNA from ZIKV strain FSS13025.

### ZIKV-binding IgG/IgM ELISA

ELISA plates (96-well, Costar) were coated with ZIKV E protein (1 μg/ml, ZIKVSU-ENV, Native Antigen) in coating buffer (0.1 M NaHCO_3_) overnight at 4°C and then blocked for 1 h at room temperature (RT) with 5% Blocker Casein in PBS (Thermo Fisher Scientific). Mouse serum samples were diluted three-fold (from 1:30 to 1:65,610) in 1% bovine serum albumin (BSA)/PBS, added to the coated wells, and incubated for 1.5 h at RT. Wells were then washed with wash buffer (0.05% Tween 20 [Promega] in PBS), and HRP-conjugated goat anti-mouse IgG Fc or goat anti-mouse IgM (1:5000 in 1% BSA/PBS) was added to each well for 1.5 h at RT. TMB chromogen solution (eBioscience) was added to the wells, the reaction was stopped by addition of sulfuric acid, and the absorbance at 450 nm was read on a Spectramax M2E microplate reader (Molecular Devices). The ZIKV-specific Ab endpoint titers were calculated as the reciprocal of the highest serum dilution that gave a reading twice the cutoff absorbance based on the negative control (BSA/PBS).

### Serum neutralization assay

Sera from naïve and ZIKV-immune mice were inactivated by incubation for 30 min at 56°C and then serially diluted and added to 96-well round-bottom plates. Some sera were incubated for 30 min with dithiothreitol (0.01 M, Sigma) prior to infection to inactivate IgM. A sufficient amount of ZIKV FSS13025 causing 7–15% of infection in U937 DC-SIGN, was added to the sera and incubated for 1 h at 37°C (titration of virus is determined for each batch of cells). U937 cells expressing dendritic cell-specific intracellular adhesion molecule-3-grabbing non-integrin (U937 DC-SIGN, ATCC) were then added at 1 x 10^5^ cells/well and the plates were incubated for 2 h at 37°C with rocking every 15 min. Cells and ZIKV FSS13025 incubated in the absence of serum served as the positive control. Plates were then centrifuged, the supernatants were aspirated, fresh RPMI medium supplemented with 10% FBS was added, and the cells were incubated for 16 h at 37°C. Finally, cells were harvested, stained with PE-conjugated anti-CD209 (DC-SIGN, clone DNC246), incubated with Cytofix/Cytoperm solution (BD Biosciences), and stained intracellularly with Alexa Fluor 647-conjugated 4G2 (to ZIKV E protein). The cells were analyzed on an LSRII flow cytometer (BD Biosciences) and the percentage of infected cells was determined using FlowJo 10.4.2 software (Tree Star, Ashland, OR). The percentage serum inhibition was calculated as 100 − (% infected cells in the presence of serum) / (% infected cells in the absence of serum) x 100.

### Intracellular cytokine staining (ICS) assay

Splenocytes were plated into 96-well round-bottom plates at 1 × 10^6^ cells/well in complete RPMI 1640 medium (containing 10% FBS, 1% penicillin/streptomycin, and 1% HEPES (all from Gibco) and stimulated with 1 μg of the indicated ZIKV peptides. After 1 h at 37°C, brefeldin A (1000X, BioLegend) was added at a 1:1000 dilution and the cells were incubated for an additional 5 h. Cells incubated with RPMI medium or with RPMI medium + PMA/ionomycin (500X) served as negative and positive controls, respectively. After incubation, the splenocytes were stained with efluor 455 (UV) viability dye, (Invitrogen) and fluorophore-conjugated Abs against mouse CD3e (clone 145-2C11, Tonbo), CD8α (clone 53–6.7, BioLegend), CD4 (clone GK1.5, Invitrogen), CD44 (clone IM7, BioLegend), CD62L (MFL-14, BioLegend) CD25 (clone PC61, BioLegend), Biotin-CD185 (clone SPRCL5, Invitrogen), CD279 (clone 29F.1A12, BioLegend), CD19 (clone ebio1D3, eBioscience), CD11a (clone M17/4, eBioscience), CD45.1 (clone A20, eBioscience), CD49d(clone R1-2, eBioscience), IgD (clone 11-26C.2a, BD Pharmingen), CD138 (clone 281.2, BioLegend) and streptavidin-conjugated BV421 (BD Pharmingen), all at 1:200 dilution. The cells were then fixed and permeabilized with Cytofix/Cytoperm (BD Bioscience) (or Fixation/Permeabilization Concentrate [eBioscience] for analysis of Treg cells) and stained with fluorophore-conjugated Abs against mouse IFNγ (clone XMG1.2, Tonbo), TNF (clone MP6-XT22, eBioscience), IL-10 (clone JES5-16E3, BioLegend), IL-2 (clone JES6-5H4, BioLegend), IL-17A (clone eBio17B7, eBioscience), IL-4 (clone 11B11, BioLegend), IL-5 (clone TRFK5, Invitrogen), granzyme B (clone NGZB, eBioscience), Bcl-6 (clone K112-91, BD Pharmingen), and/or FOXP3 (clone FJK-16S, eBioscience). Data were collected on an LSR II (BD Bioscience) and analyzed using FlowJo software.

### Peptide immunization

Five-week-old *LysMCre*^+^*Ifnar1*^fl/fl^ and *Ifnar1*^*-/-*^ mice were injected subcutaneously with 100 μg each of six immunodominant peptides (E_346-360,_ E_644-658_, NS3_1740-1754_, NS4B_2480-2494_, NS5_2604-2618_, NS5_2738-2752_) in complete Freund’s adjuvant. Two weeks later, the mice were boosted by injection of the same peptides in incomplete Freund’s adjuvant. Fourteen days later, the mice were infected retro-orbitally with 10^5^ FFU ZIKV. Three days post-infection, organs were collected and infectious ZIKV particles were quantified using FFA.

### In vivo cytotoxicity assay

To prepare target cells, splenocytes were harvested from naïve donor mice (C57BL6, CD45.1) and incubated for 3 h at 37°C with a pool of H2-IA^b^-restricted peptides (E_346-360,_ E_644-658_, NS3_1740-1754_, NS4B_2480-2494_, NS5_2604-2618_, NS5_2738-2752_) or DMSO. Cells were then washed and labeled with CSFE (Invitrogen) in PBS/0.1% BSA for 10 min at 37°C. To distinguish between target cells, cells incubated with DMSO and ZIKV peptides were labeled with 100 nM CSFE (low) and 1 μM CSFE (high), respectively. Cells were then washed and 5 × 10^6^ each of CSFE-low and CSFE-high target cells were mixed and injected RO into recipient *LysMCre*^+^*Ifnar1*^fl/fl^ mice that had been infected retro-orbitally with 10^4^ FFU ZIKV FSS13025, 10^4^ FFU ZIKV MR766, or vehicle (10% FBS/PBS; mock-infected). Twelve hours later, splenocytes were harvested from the recipient mice and the number of CSFE-labeled cells was quantified by flow cytometry. The specific percentage killing was calculated as follows: 100 − ([percentage specific (CSFE-high) target cells present in infected mice]/[percentage non-specific (CSFE-low) target cells present in infected mice]/[percentage specific (CSFE-high) target cells present in mock-infected mice]/[percentage non-specific (CSFE-low) target cells present in mock-infected mice] x 100).

### Clinical scoring of disease

Mice were weighed on the day of infection and then weighed and scored for disease daily post-infection. Clinical features were based on a 7-point scale: 1, healthy; 2, slightly ruffled coat around head and neck; 3, ruffled coat over the entire body; 4, severely ruffled coat and slightly closed eyes; 5, sick with closed eyes and slow movement (mice were euthanized); 6, no movement and slow breathing; 7 dead. Weight loss was calculated by comparison with the weight on the day of infection.

### Statistical analyses

All data were analyzed with Prism software, version 7.0 (GraphPad Software). Results are expressed as the mean ± standard errors. A non-parametric Mann–Whitney test was used to compare differences between two groups, and the Wilcoxon test was used to compare two parameters from the same group. One-way or two-way ANOVA or a Kruskal–Wallis test was used to compare more than two groups. *P* < 0.05 was considered significant.

## Supporting information

S1 Fig (Related to Fig 1). Immunodominant Th1 epitopes do not induce Th2 or Th17 cell responses. *LysMCre*^+^*Ifnar1*^fl/fl^ C57BL/6 mice were infected retro-orbitally with 10^4^ FFU of ZIKV strains MR766 or FSS13025. At day 7 post-infection, splenocytes were prepared and stimulated *in vitro* with one of the indicated immunodominant ZIKV epitopes in the presence of brefeldin A for 5 h. The frequency of cells producing (**A**) IL-4, (**B**) IL-5, or (**C**) IL-17A was assessed by ICS. All experiments were performed twice. Data are the mean ± SEM of *n* = 4 mice per group. Cells incubated with DMSO or PMA/ionomycin served as negative and positive controls, respectively. The dotted line corresponds to the average frequency of cytokine-producing cells from mock-infected mice.
(TIFF)

S2 Fig (Related to Fig 2). Induction of T_FH_ and Treg cells after ZIKV infection. *LysMCre*^+^*Ifnar1*^fl/fl^ C57BL/6 mice were infected retro-orbitally with 10^4^ FFU of ZIKV strain FSS13025 or mock-infected by injection of vehicle alone (10% FBS/PBS). At day 7 post-infection, splenocytes were processed for flow cytometric analysis. (**A and B**) Gating strategy used to analyze (**A**) CXCR5^+^PD1^+^ T_FH_ cells and (**B**) Foxp3^+^CD25^+^ Treg cells. (**C and D**) Numbers of T_FH_ (**C**) and Treg cells (**D**) among CD4^+^CD44^+^ T cells. Mean ± SEM of *n* = 4 mock-infected and *n* = 6 ZIKV-infected mice. (**E**) Representative contour plot showing the frequency of IFNγ- and IL-10-producing CD44^+^CD4^+^ T cells from the day 7 post-infection splenocytes prepared and stimulated *in vitro* with ZIKV epitope E_644-658_ in the presence of brefeldin A for 5 h. **P < 0.01 by the Mann–Whitney *U* test.
(TIFF)

S3 Fig (Related to Fig 3). Ab production and CD8^+^ T cell activation in response to primary ZIKV infection in mice depleted of CD4^+^ T cells. *LysMCre*^+^*Ifnar1*^fl/fl^ C57BL/6 mice were treated with anti-CD4 or isotype control Ab on days −3 and −1 prior to and every 2 days after retro-orbital infection with 10^5^ FFU of ZIKV strain FSS13025. (**A–C**) On day 10 post-infection, serum samples were analyzed for (**A**) anti-ZIKV E IgM and (**B**) anti-ZIKV E IgG by ELISA or (**C**) neutralizing activity using a U937 DC-SIGN cell-based flow cytometric assay. (**D–G**) On day 7 post-infection, splenocytes were prepared and stimulated *in vitro* with the class I-restricted ZIKV epitopes PrM_169-177,_ E_297-305,_ and NS5_2783-2792_ for 4 h. The number of total CD8^+^CD3^+^ cells (**D**), CD44^high^CD62L^low^CD8^+^ T cells (**E**), IFNγ-producing CD8^+^ T cells (**F**), and IFNγ + TNF-producing CD8^+^ T cells (**G**) were analyzed by flow cytometry. Data are the mean ± SEM of *n* = 4 mice per group. Isotype control and anti-CD4 groups were compared using the Mann–Whitney *U* test. No significant differences were detected.
(TIFF)

S4 Fig (Related to Fig 3). CD4^+^ T cell roles in the Ab and CD8^+^ T cell responses and viral control after intrafootpad infection with ZIKV. *LysMCre*^+^*Ifnar1*^fl/fl^ C57BL/6 mice were treated with a depleting anti-CD4 Ab or isotype control Ab on days −3 and −1 prior to and every 2 days after intrafootpad infection with 10^5^ FFU of ZIKV FSS13025. (**A–C**) Sera were collected on day 7 post-infection to measure anti-ZIKV IgM (**A**) and IgG (**B**) titers by ZIKV E-specific ELISA and (**C**) ZIKV neutralizing activity using a U937 DC-SIGN cell-based flow cytometric assay. Mean ± SEM of *n* = 8 isotype control mice and *n* = 7 anti-CD4-treated mice. (**D and E**) Splenocytes were collected on day 7 post-infection and analyzed by flow cytometry for the percentage of CD138^+^IgD^−^ plasma cells (**D**) or GL7^+^Fas^+^ germinal center B cells (**E**). (**F**) CD8^+^ T cell were stimulated with the class I-binding ZIKV peptides PrM_169-177_ or NS5_2783-2792_ and analyzed for the percentage of IFNγ-producing (**F**) or IFNγ + TNF-producing (**G**) CD8^+^ T cells. Data are the mean ± SEM of *n* = 8 isotype control mice and *n* = 7 anti-CD4-treated mice. (**H**) Serum, brain, and testes were harvested on day 7 post-infection and infectious ZIKV titers were determined using a focus-forming assay. Data are the mean ± SEM of *n* = 8 (serum and brain) or *n* = 4 (testes) for isotype control Ab-treated mice and *n* = 5 for anti-CD4-treated mice. ***P < 0.001 by the Mann–Whitney *U* test. Data were pooled from two independent experiments.
(TIFF)

S5 Fig (Related to Fig 4). CD4^+^ T cell responses after secondary ZIKV infection in *LysMCre*^+^*Ifnar1*^fl/fl^ mice. *LysMCre*^+^*Ifnar1*^fl/fl^ C57BL/6 mice were infected with 10^4^ FFU of ZIKV FSS13025 or vehicle (10% FBS-PBS) for 30 days, treated with a depleting anti-CD4 Ab (*n* = 8) or isotype control Ab (*n* = 9) on days −3 and −1, and challenged with 10^3^ FFU of ZIKV FSS13025 on day 0. (**A and B**) Splenocytes were collected on day 3 after secondary ZIKV challenge and analyzed by flow cytometry for the percentage of (**A**) CD138^+^IgD^−^ plasma cells and (**B**) GL7^+^Fas^+^ germinal center B cells. (**C and D**) CD8^+^ T cells were stimulated with the class I-binding ZIKV peptides (**C**) PrM_169-177_ or (**D**) NS5_2783-2792_ and analyzed for the presence of IFNγ- or IFNγ^+^ TNF^+^-producing cells. (**E and F)** Splenocytes were analyzed by flow cytometry for the percentage of (**E**) T_FH_ cells and (**F**) Treg cells. (**G**) Splenocytes were stimulated with E_644-658_ peptide for 6 h and analyzed for the production of IFNγ-, IFNγ + TNF-, and IL-2-producing cells by flow cytometry. Data are the mean ± SEM of 10 mice/group. *P < 0.05, **P < 0.01 by the Mann–Whitney *U* test. Data were pooled from two independent experiments.
(TIFF)

S6 Fig (Related to Fig 4). No role for CD4^+^ T cells in protecting against lethal ZIKV challenge in *Ifnar1*^−/−^ mice immunized with ZIKV peptides. (**A–D**) Five-week-old *Ifnar1*^−/−^ C57BL/6 mice were immunized subcutaneously with a mixture of six immunodominant ZIKV peptides (ZIKV, *n* = 13) or DMSO (Mock, *n* = 12) on day 0, boosted with the same peptides on day 14, and infected with 10^3^ FFU of ZIKV FSS13025 on day 28. (**A**) Mortality. (**B**) Percentage weight loss *vs*. day 0. (**C and D**) Clinical disease scores in mock-infected (**C**) and ZIKV-infected (**D**) mice. Data are the mean ± SEM. *P < 0.05. Mann–Whitney *U* test was used to compare weight loss between groups at each time point, and Gehan–Breslow Wilcoxon test was used to compare survival. Data were pooled from two independent experiments.
(TIFF)

S7 Fig (Related to Fig 3–4). CD4^+^ T cell depletion prior to lethal primary or secondary ZIKV challenge in *Ifnar1*^−/−^ mice. (**A–D**). Five-week old *Ifnar1*^−/−^ C57BL/6 mice were treated with anti-CD4 Ab or isotype control Ab on days −3 and −1 prior to infection with 10^2^ FFU of ZIKV FSS13025. (**A**) Mortality. (**B**) Percentage weight loss *vs*. day 0. (**C and D**) Clinical disease scores in isotype control Ab-treated (**C**) and anti-CD4 Ab-treated (**D**) mice. (**E and F**) Five-week old *Ifnar1*^−/−^ C57BL/6 mice were treated with anti-CD4 Ab (ZIKV-immune α-CD4, *n* = 7) or isotype control Ab (ZIKV-immune isotype, *n* = 6) on days −3 and −1 prior to and then every week after infection with 10^1^ FFU of ZIKV FSS13025. On day 30 post-infection, both groups and a group of age-matched mice (Mock-immune, *n* = 7) were infected with 10^3^ FFU of ZIKV FSS13025. (**E**) Mortality. (**F**) Percentage weight loss. Data are the mean ± SEM. **P < 0.01. Mann–Whitney *U* test was used to compare weight loss between ZIKV-immune isotype and ZIKV-immune anti-CD4 groups at each time point. Gehan–Breslow Wilcoxon test was used to compare survival. Data were pooled from two independent experiments.
(TIFF)

S8 Fig (Related to Fig 5). Characterization of CD4^+^ T cell subsets in iliac lymph nodes after intravaginal infection of *LysMCre*^+^*Ifnar1*^fl/fl^ mice with ZIKV. Eight-week-old female *LysMCre*^+^*Ifnar1*^fl/fl^ C57BL/6 mice were treated with progesterone to induce a diestrus-like phase and intravaginally administered 10% FBS/PBS (mock-infected) or 10^5^ of ZIKV strain FSS13025. On day 10 post-infection, cells were isolated from the iliac lymph nodes and stimulated *in vitro* with the CD4^+^ T cell epitope E_644-658_ in the presence of brefeldin A. Cells were then analyzed by flow cytometry for the frequency of (**A**) CXCR5^+^PD1^+^CD44^+^CD4^+^ T_FH_ cells, (**B**) FoxP3^+^CD25^+^CD44^+^CD4^+^ Treg cells, and (**C**) IL-10-producing CD44^+^CD4^+^ T cells. Data are the mean ± SEM of *n* = 3–4 mock-infected mice and *n* = 5–6 ZIKV-infected mice. *P < 0.05, **P < 0.01 by the Mann–Whitney *U* test.
(TIFF)

S9 Fig (Related to Fig 5). Characterization of the CD8+ T cell response to primary intravaginal infection of *LysMCre^+^Ifnar1^fl/fl^* mice with ZIKV. Eight-week-old female *LysMCre*^+^*Ifnar1*^fl/fl^ C57BL/6 mice were treated with 2 mg of progesterone to induce a synchronized diestrus-like phase. Three days post-treatment, mice were intravaginally administered 10% FBS/PBS (mock-infected) or 10^5^ of ZIKV strain FSS13025. At day 10 post-infection, splenocytes were stimulated *in vitro* with the ZIKV CD8^+^ T cell epitope E_297-305_ and analyzed by flow cytometry for the percentage of (**A**) CD8^+^CD44^high^CD62L^−^ and CD8^+^CD44^high^CD62L^+^ cells, (**B**) antigen-experienced (CD11a^high^) CD8^+^ T cells, and (**C**) IFNγ- and IFNγ + TNF-producing CD8^+^ T cells. Data are the mean ± SEM of *n* = 4 mice per group. *P < 0.05 by the Mann–Whitney *U* test.
(TIFF)

S10 Fig (Related to Fig 6). CD4^+^ T cell response to intravaginal ZIKV infection in mice depleted of CD8^+^ T cells. Eight-week-old *LysMCre*^+^*Ifnar1*^fl/fl^ C57BL/6 mice were administered anti-CD8 (*n* = 8) or an isotype control Ab (*n* = 8) on days −3 and −1 prior to and every 2 days after intravaginal administration of 10% FBS/PBS (mock-infected) or 10^5^ of ZIKV strain FSS13025. On day 10 post-infection, spleen and iliac lymph nodes were collected, and single-cell suspensions were stimulated *in vitro* with the CD4^+^ T cell ZIKV epitope E_644-658_. Cells were analyzed by flow cytometry for the (**A**) frequency and (**B**) total number of IFNγ- and IFNγ + TNF-producing CD44^+^CD4^+^ T cells in the spleen and (**C**) frequency of IFNγ- and IFNγ + TNF-producing CD44^+^CD4^+^ T cells in the iliac lymph nodes. Data are the mean ± SEM of *n* = 8 mice per group. **P < 0.01, ***P < 0.001 by the Mann–Whitney *U* test.
(TIFF)

S11 Fig (Related to Fig 6). Increased mortality in *Ifnar1*^−/−^ mice that were depleted of CD4^+^ T cells prior to intravaginal ZIKV challenge. (**A–D**). Eight-week-old *Ifnar1*^−/−^ C57BL/6 mice were treated with progesterone and anti-CD4 Ab (*n* = 9) or isotype control Ab (*n* = 9) on days −3 and −1 prior to IVag infection with 10^6^ FFU of ZIKV FSS13025. (**A**) Mortality. (**B**) Percentage weight loss *vs*. day 0. (**C and D**) Clinical disease score for the isotype control Ab-treated group (**C**) and anti-CD4 Ab-treated group (**D**) were monitored daily and represented. Data are the mean ± SEM. **P < 0.01. Mann–Whitney *U* test was used to compare weight loss at each time point, and Gehan–Breslow Wilcoxon test was used to compare survival. Data were pooled from two independent experiments.
(TIFF)
