# Correction: CD4^+^ T cells promote humoral immunity and viral control during Zika virus infection

**DOI:** 10.1371/journal.ppat.1007821

**Published:** 2019-05-28

**Authors:** Annie Elong Ngono, Matthew P. Young, Maximilian Bunz, Zhigang Xu, Sararat Hattakam, Edward Vizcarra, Jose Angel Regla-Nava, William W. Tang, Montarop Yamabhai, Jinsheng Wen, Sujan Shresta

In the Funding statement, one of the grant numbers is missing. The correct Funding statement is as follows: This work was funded by NIAID/NIH grants R01 AI116813, R21 NS100477, R21 AI140063, and R01 NS106387 to SS and the Chiba-UCSD Center for Mucosal Immunology, Allergy and Vaccine Development. The funders had no role in study design, data collection and analysis, decision to publish, or preparation of the manuscript.
