# Supplementary figures and images for: CD4+ T cells promote humoral immunity and viral control during Zika virus infection
Source: PLoS Pathog. 2019 Jan 24;15(1):e1007474. doi: 10.1371/journal.ppat.1007474 (PMC6345435; doi:10.1371/journal.ppat.1007474)

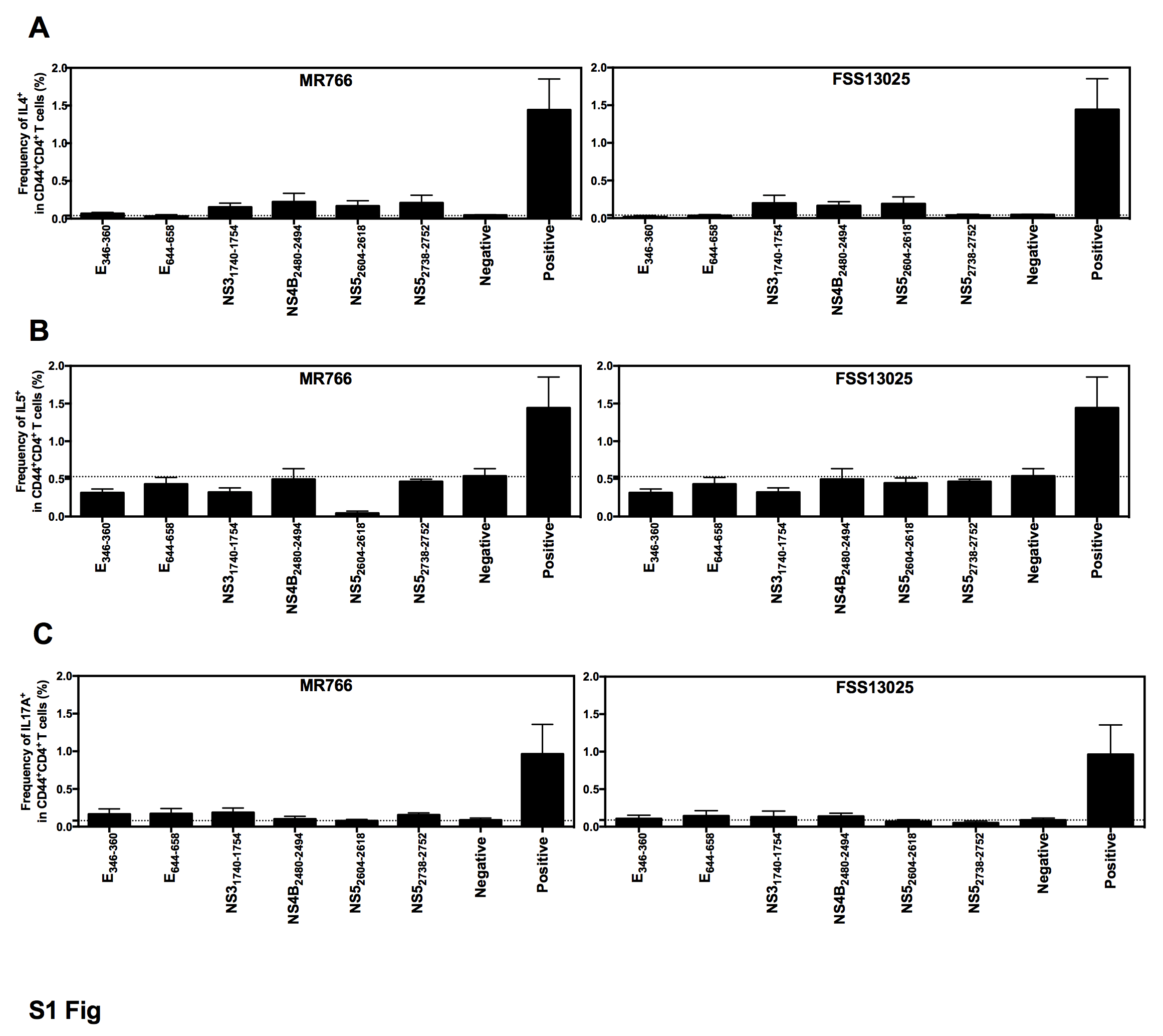

Supplement: S1 Fig — LysMCre+Ifnar1fl/fl C57BL/6 mice were infected retro-orbitally with 104 FFU of ZIKV strains MR766 or FSS13025. At day 7 post-infection, splenocytes were prepared and stimulated in vitro with one of the indicated immunodominant ZIKV epitopes in the presence of brefeldin A for 5 h. The frequency of cells producing (A) IL-4, (B) IL-5, or (C) IL-17A was assessed by ICS. All experiments were performed twice. Data are the mean ± SEM of n = 4 mice per group. Cells incubated with DMSO or PMA/ionomycin served as negative and positive controls, respectively. The dotted line corresponds to the average frequency of cytokine-producing cells from mock-infected mice. (TIFF) [file ppat.1007474.s001.tiff]

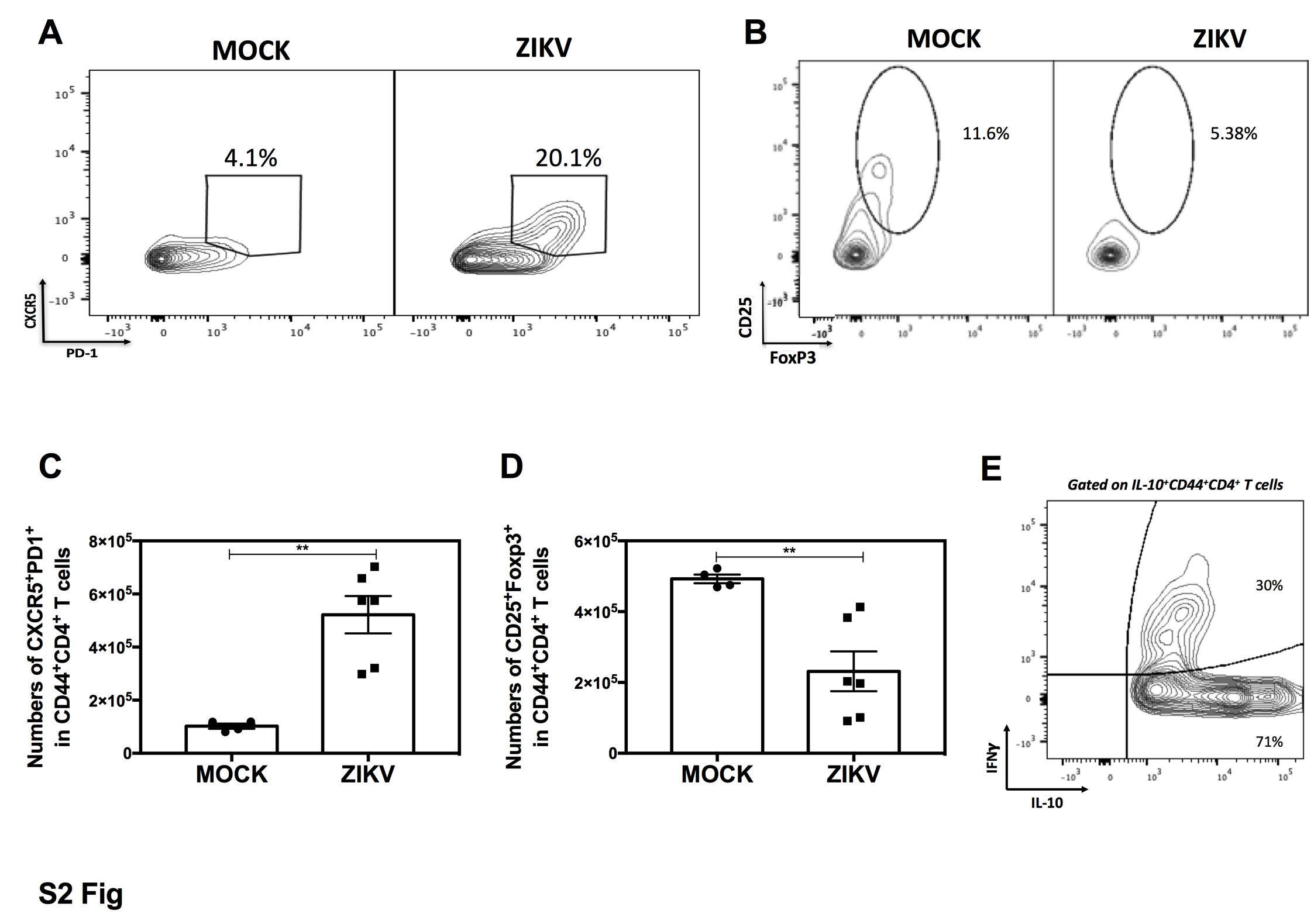

Supplement: S2 Fig — LysMCre+Ifnar1fl/fl C57BL/6 mice were infected retro-orbitally with 104 FFU of ZIKV strain FSS13025 or mock-infected by injection of vehicle alone (10% FBS/PBS). At day 7 post-infection, splenocytes were processed for flow cytometric analysis. (A and B) Gating strategy used to analyze (A) CXCR5+PD1+ TFH cells and (B) Foxp3+CD25+ Treg cells. (C and D) Numbers of TFH (C) and Treg cells (D) among CD4+CD44+ T cells. Mean ± SEM of n = 4 mock-infected and n = 6 ZIKV-infected mice. (E) Representative contour plot showing the frequency of IFNγ- and IL-10-producing CD44+CD4+ T cells from the day 7 post-infection splenocytes prepared and stimulated in vitro with ZIKV epitope E644-658 in the presence of brefeldin A for 5 h. **P < 0.01 by the Mann–Whitney U test. (TIFF) [file ppat.1007474.s002.tiff]

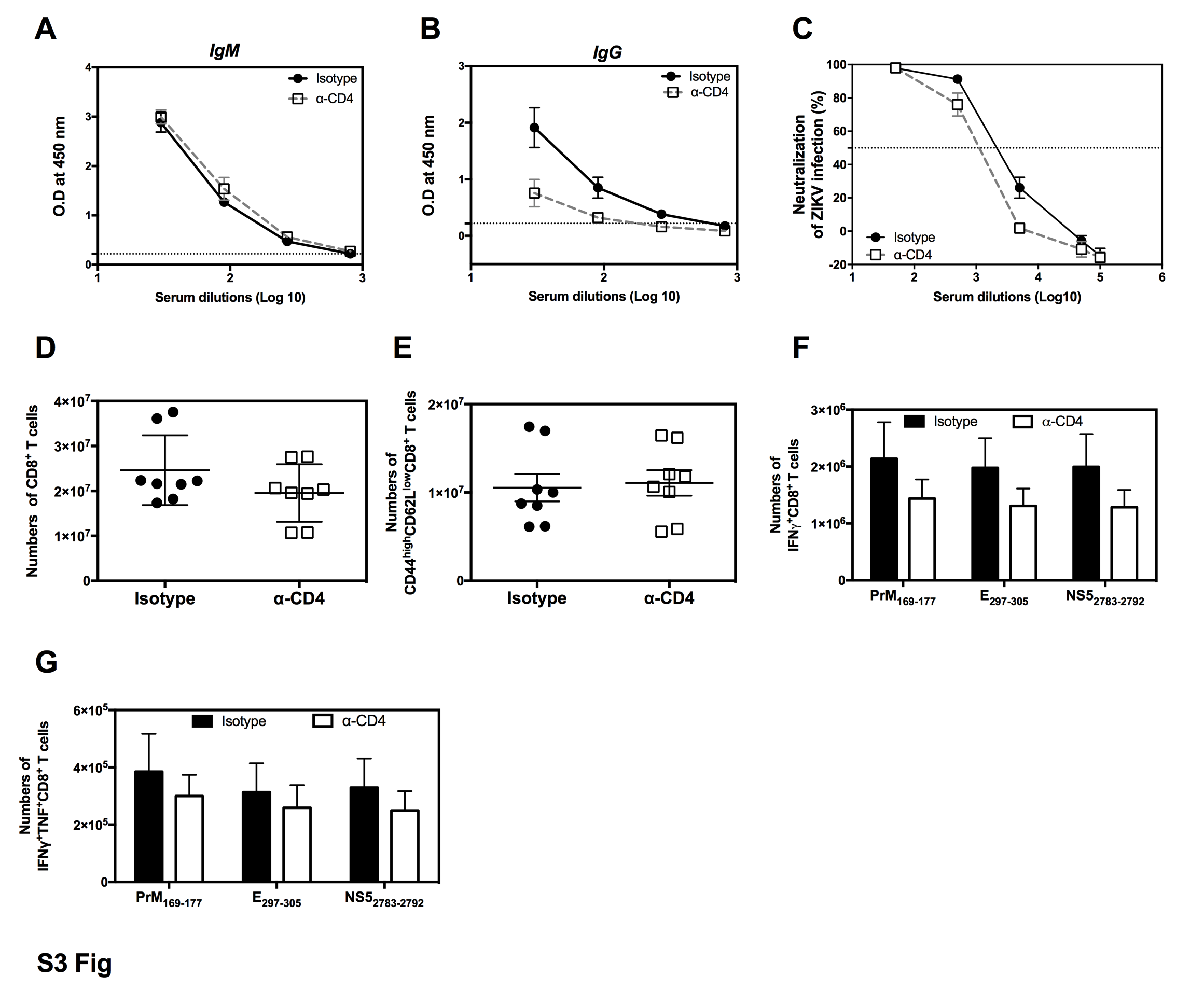

Supplement: S3 Fig — LysMCre+Ifnar1fl/fl C57BL/6 mice were treated with anti-CD4 or isotype control Ab on days −3 and −1 prior to and every 2 days after retro-orbital infection with 105 FFU of ZIKV strain FSS13025. (A–C) On day 10 post-infection, serum samples were analyzed for (A) anti-ZIKV E IgM and (B) anti-ZIKV E IgG by ELISA or (C) neutralizing activity using a U937 DC-SIGN cell-based flow cytometric assay. (D–G) On day 7 post-infection, splenocytes were prepared and stimulated in vitro with the class I-restricted ZIKV epitopes PrM169-177, E297-305, and NS52783-2792 for 4 h. The number of total CD8+CD3+ cells (D), CD44highCD62LlowCD8+ T cells (E), IFNγ-producing CD8+ T cells (F), and IFNγ + TNF-producing CD8+ T cells (G) were analyzed by flow cytometry. Data are the mean ± SEM of n = 4 mice per group. Isotype control and anti-CD4 groups were compared using the Mann–Whitney U test. No significant differences were detected. (TIFF) [file ppat.1007474.s003.tiff]

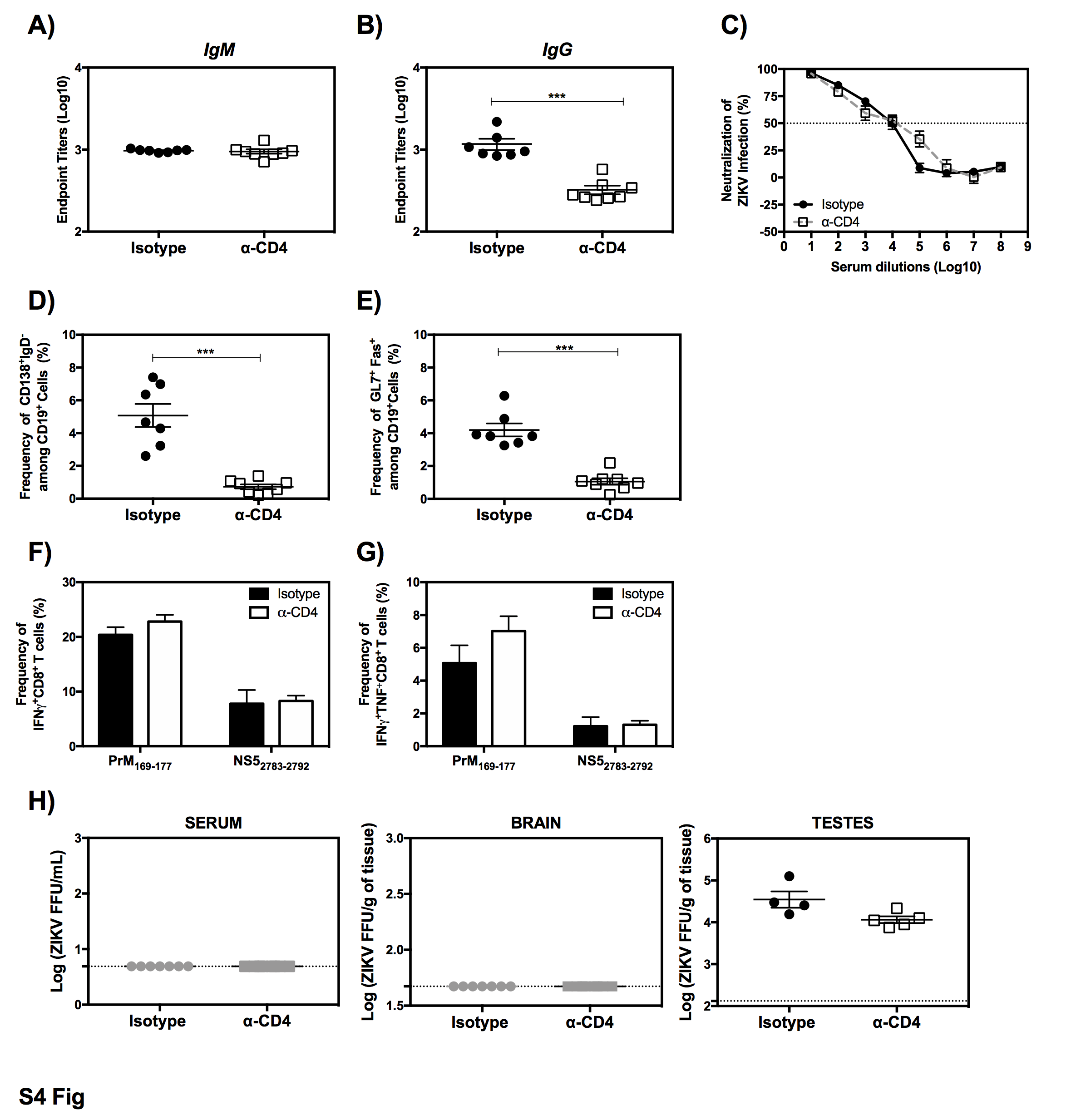

Supplement: S4 Fig — LysMCre+Ifnar1fl/fl C57BL/6 mice were treated with a depleting anti-CD4 Ab or isotype control Ab on days −3 and −1 prior to and every 2 days after intrafootpad infection with 105 FFU of ZIKV FSS13025. (A–C) Sera were collected on day 7 post-infection to measure anti-ZIKV IgM (A) and IgG (B) titers by ZIKV E-specific ELISA and (C) ZIKV neutralizing activity using a U937 DC-SIGN cell-based flow cytometric assay. Mean ± SEM of n = 8 isotype control mice and n = 7 anti-CD4-treated mice. (D and E) Splenocytes were collected on day 7 post-infection and analyzed by flow cytometry for the percentage of CD138+IgD− plasma cells (D) or GL7+Fas+ germinal center B cells (E). (F) CD8+ T cell were stimulated with the class I-binding ZIKV peptides PrM169-177 or NS52783-2792 and analyzed for the percentage of IFNγ-producing (F) or IFNγ + TNF-producing (G) CD8+ T cells. Data are the mean ± SEM of n = 8 isotype control mice and n = 7 anti-CD4-treated mice. (H) Serum, brain, and testes were harvested on day 7 post-infection and infectious ZIKV titers were determined using a focus-forming assay. Data are the mean ± SEM of n = 8 (serum and brain) or n = 4 (testes) for isotype control Ab-treated mice and n = 5 for anti-CD4-treated mice. ***P < 0.001 by the Mann–Whitney U test. Data were pooled from two independent experiments. (TIFF) [file ppat.1007474.s004.tiff]

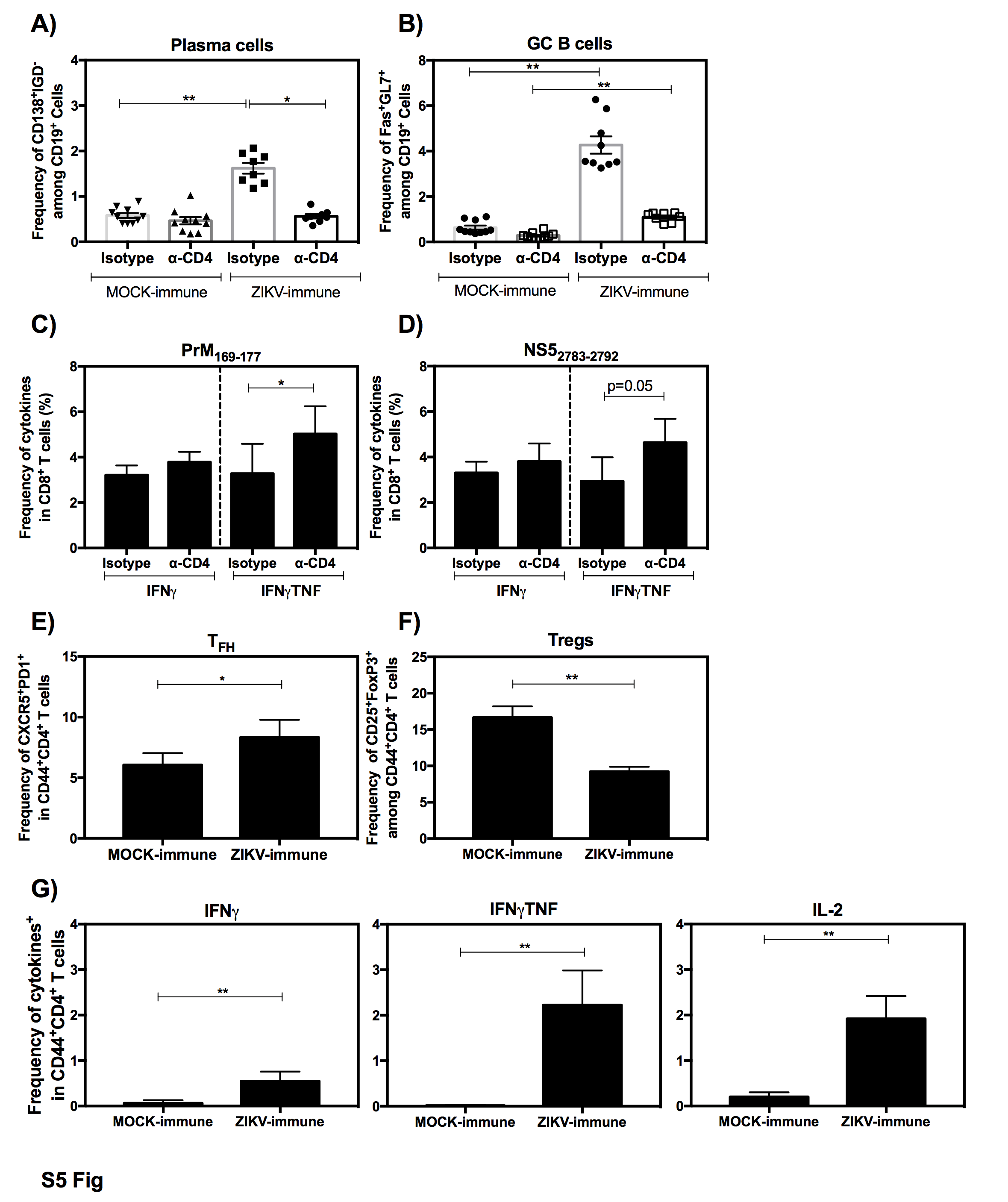

Supplement: S5 Fig — LysMCre+Ifnar1fl/fl C57BL/6 mice were infected with 104 FFU of ZIKV FSS13025 or vehicle (10% FBS-PBS) for 30 days, treated with a depleting anti-CD4 Ab (n = 8) or isotype control Ab (n = 9) on days −3 and −1, and challenged with 103 FFU of ZIKV FSS13025 on day 0. (A and B) Splenocytes were collected on day 3 after secondary ZIKV challenge and analyzed by flow cytometry for the percentage of (A) CD138+IgD− plasma cells and (B) GL7+Fas+ germinal center B cells. (C and D) CD8+ T cells were stimulated with the class I-binding ZIKV peptides (C) PrM169-177 or (D) NS52783-2792 and analyzed for the presence of IFNγ- or IFNγ+ TNF+-producing cells. (E and F) Splenocytes were analyzed by flow cytometry for the percentage of (E) TFH cells and (F) Treg cells. (G) Splenocytes were stimulated with E644-658 peptide for 6 h and analyzed for the production of IFNγ-, IFNγ + TNF-, and IL-2-producing cells by flow cytometry. Data are the mean ± SEM of 10 mice/group. *P < 0.05, **P < 0.01 by the Mann–Whitney U test. Data were pooled from two independent experiments. (TIFF) [file ppat.1007474.s005.tiff]

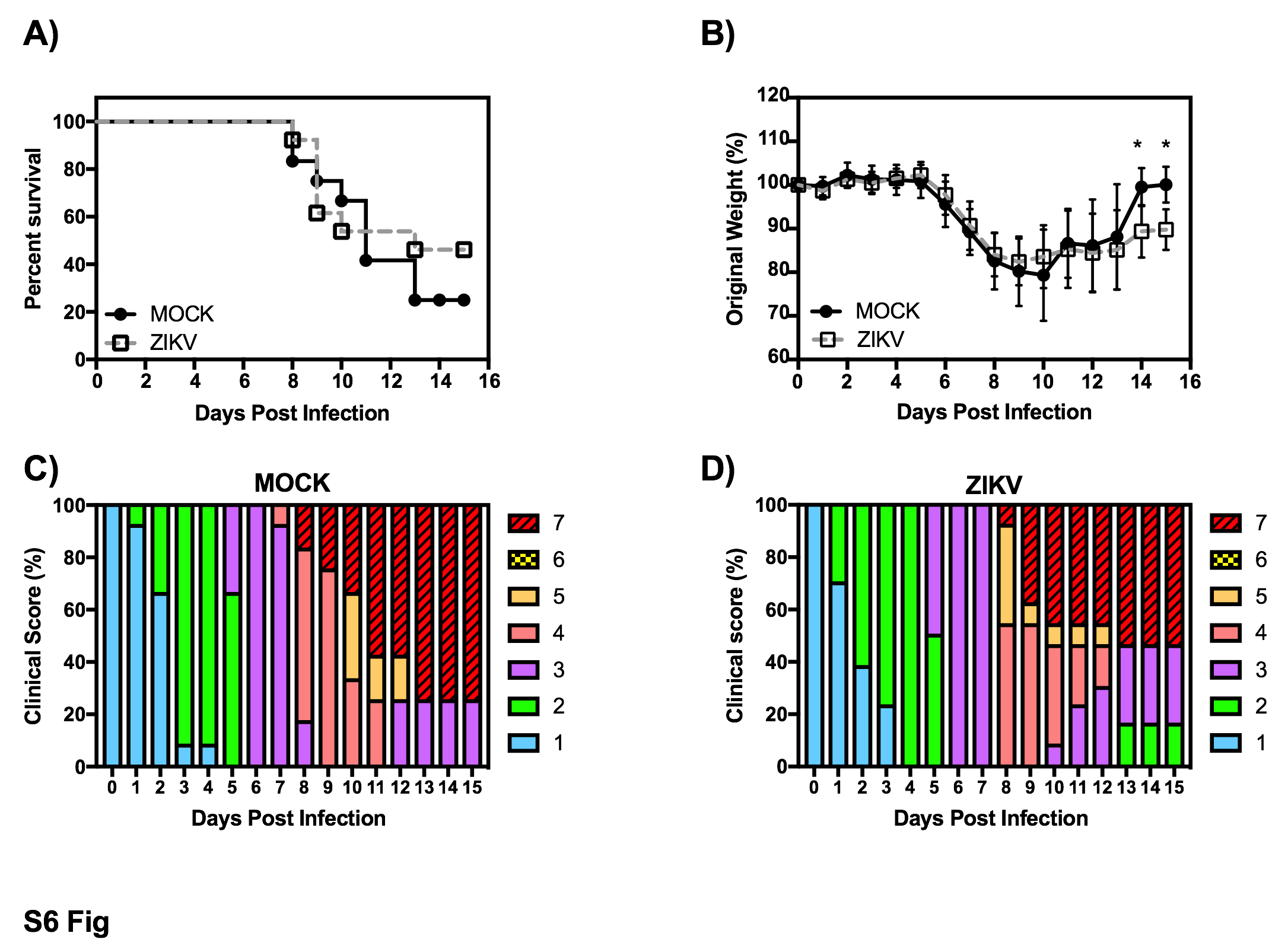

Supplement: S6 Fig — (A–D) Five-week-old Ifnar1−/− C57BL/6 mice were immunized subcutaneously with a mixture of six immunodominant ZIKV peptides (ZIKV, n = 13) or DMSO (Mock, n = 12) on day 0, boosted with the same peptides on day 14, and infected with 103 FFU of ZIKV FSS13025 on day 28. (A) Mortality. (B) Percentage weight loss vs. day 0. (C and D) Clinical disease scores in mock-infected (C) and ZIKV-infected (D) mice. Data are the mean ± SEM. *P < 0.05. Mann–Whitney U test was used to compare weight loss between groups at each time point, and Gehan–Breslow Wilcoxon test was used to compare survival. Data were pooled from two independent experiments. (TIFF) [file ppat.1007474.s006.tiff]

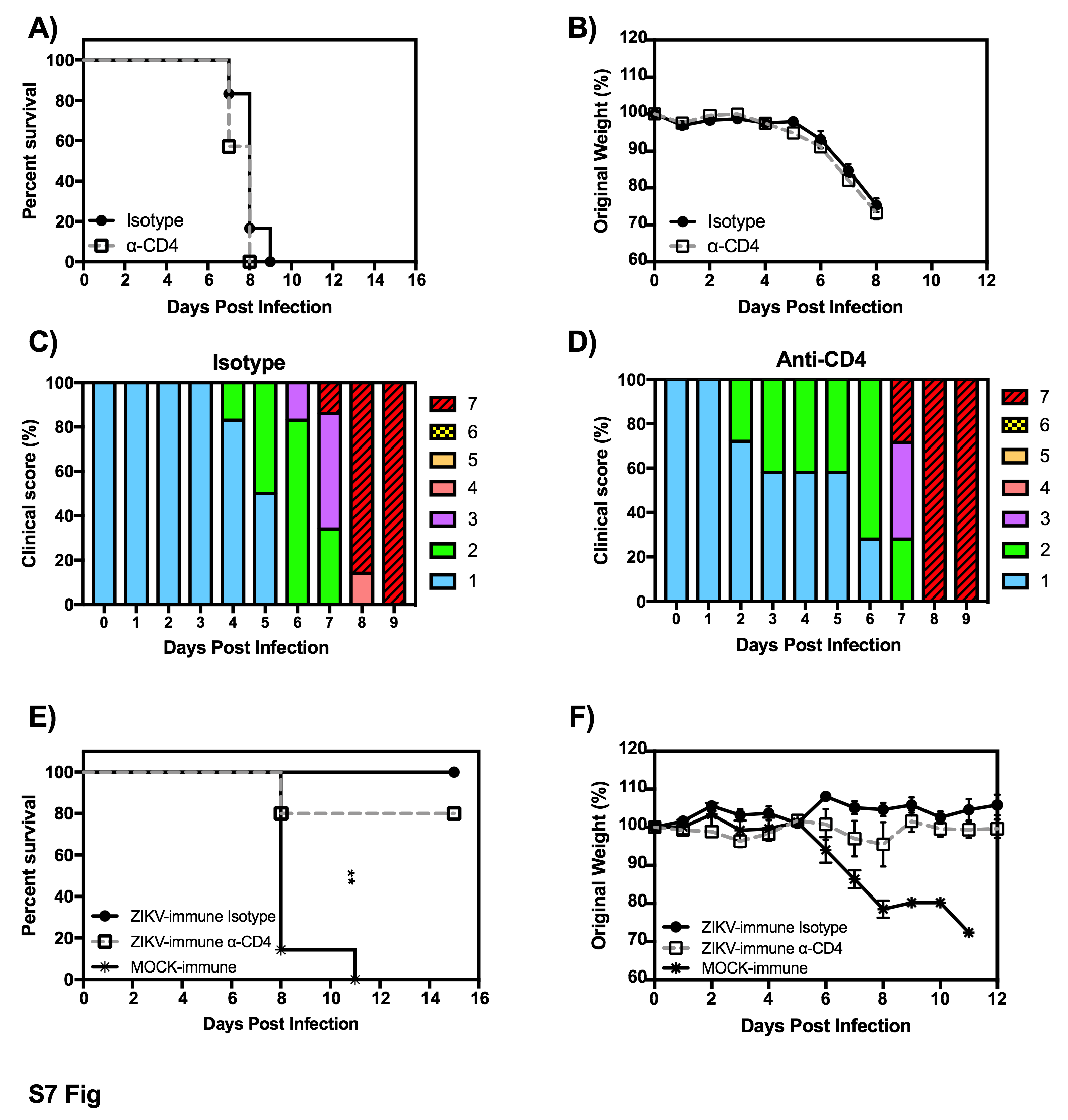

Supplement: S7 Fig — (A–D). Five-week old Ifnar1−/− C57BL/6 mice were treated with anti-CD4 Ab or isotype control Ab on days −3 and −1 prior to infection with 102 FFU of ZIKV FSS13025. (A) Mortality. (B) Percentage weight loss vs. day 0. (C and D) Clinical disease scores in isotype control Ab-treated (C) and anti-CD4 Ab-treated (D) mice. (E and F) Five-week old Ifnar1−/− C57BL/6 mice were treated with anti-CD4 Ab (ZIKV-immune α-CD4, n = 7) or isotype control Ab (ZIKV-immune isotype, n = 6) on days −3 and −1 prior to and then every week after infection with 101 FFU of ZIKV FSS13025. On day 30 post-infection, both groups and a group of age-matched mice (Mock-immune, n = 7) were infected with 103 FFU of ZIKV FSS13025. (E) Mortality. (F) Percentage weight loss. Data are the mean ± SEM. **P < 0.01. Mann–Whitney U test was used to compare weight loss between ZIKV-immune isotype and ZIKV-immune anti-CD4 groups at each time point. Gehan–Breslow Wilcoxon test was used to compare survival. Data were pooled from two independent experiments. (TIFF) [file ppat.1007474.s007.tiff]

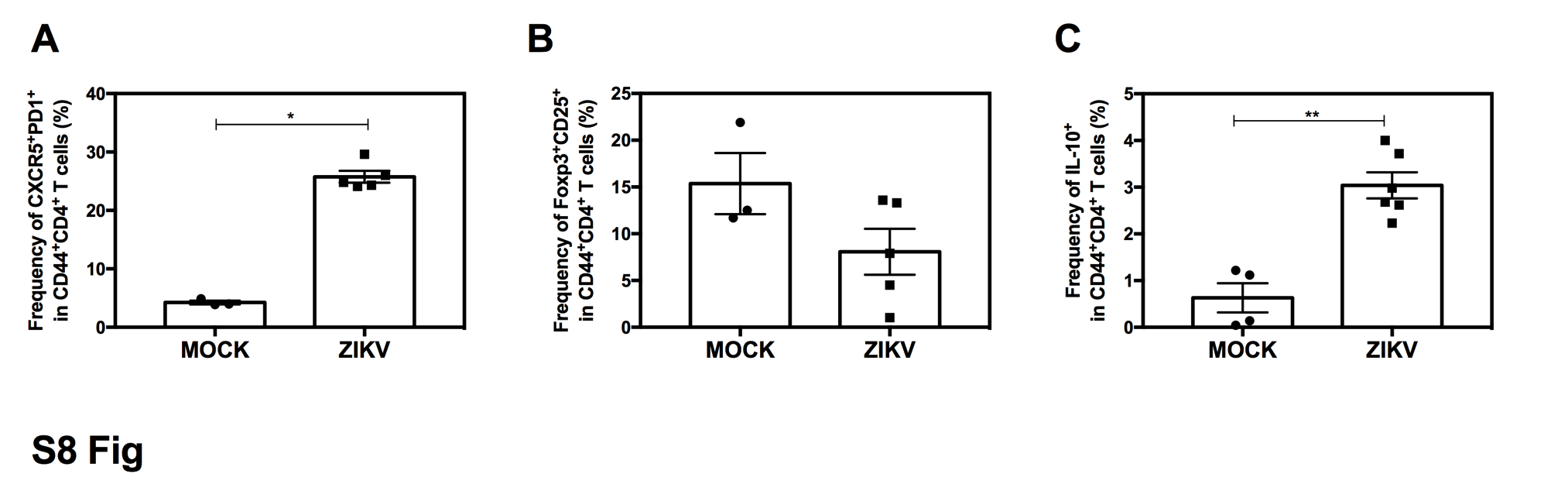

Supplement: S8 Fig — Eight-week-old female LysMCre+Ifnar1fl/fl C57BL/6 mice were treated with progesterone to induce a diestrus-like phase and intravaginally administered 10% FBS/PBS (mock-infected) or 105 of ZIKV strain FSS13025. On day 10 post-infection, cells were isolated from the iliac lymph nodes and stimulated in vitro with the CD4+ T cell epitope E644-658 in the presence of brefeldin A. Cells were then analyzed by flow cytometry for the frequency of (A) CXCR5+PD1+CD44+CD4+ TFH cells, (B) FoxP3+CD25+CD44+CD4+ Treg cells, and (C) IL-10-producing CD44+CD4+ T cells. Data are the mean ± SEM of n = 3–4 mock-infected mice and n = 5–6 ZIKV-infected mice. *P < 0.05, **P < 0.01 by the Mann–Whitney U test. (TIFF) [file ppat.1007474.s008.tiff]

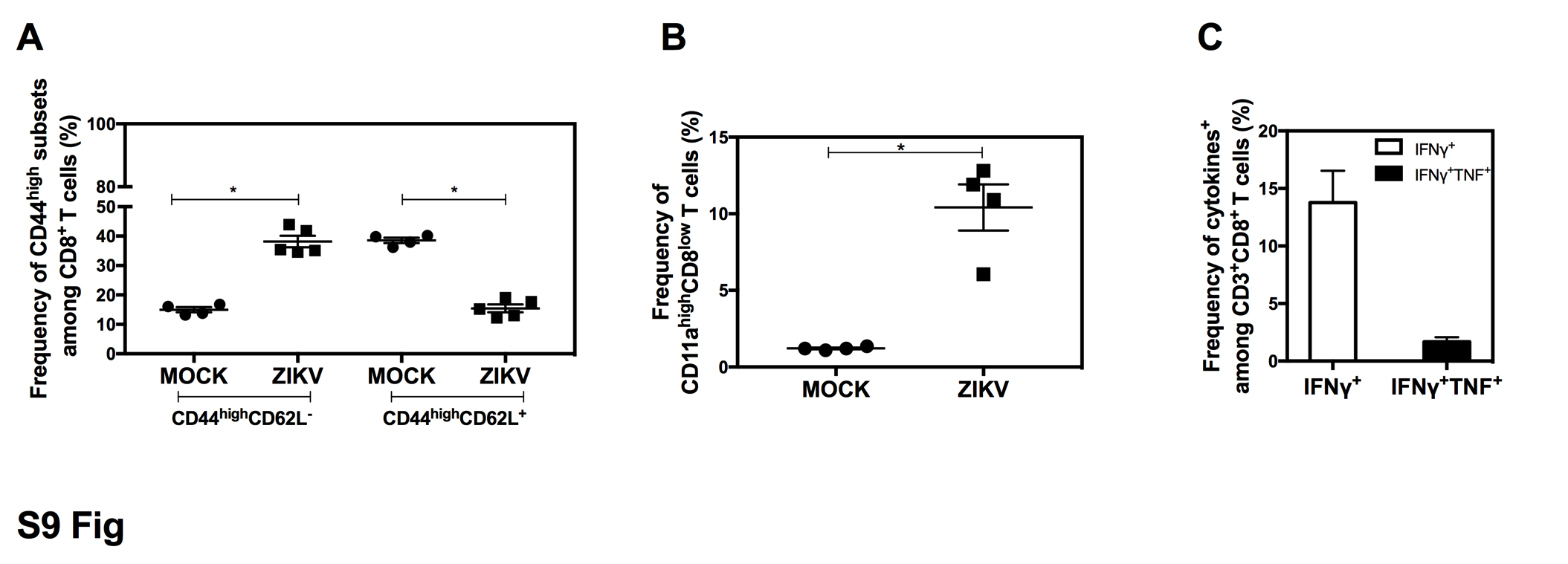

Supplement: S9 Fig — Eight-week-old female LysMCre+Ifnar1fl/fl C57BL/6 mice were treated with 2 mg of progesterone to induce a synchronized diestrus-like phase. Three days post-treatment, mice were intravaginally administered 10% FBS/PBS (mock-infected) or 105 of ZIKV strain FSS13025. At day 10 post-infection, splenocytes were stimulated in vitro with the ZIKV CD8+ T cell epitope E297-305 and analyzed by flow cytometry for the percentage of (A) CD8+CD44highCD62L− and CD8+CD44highCD62L+ cells, (B) antigen-experienced (CD11ahigh) CD8+ T cells, and (C) IFNγ- and IFNγ + TNF-producing CD8+ T cells. Data are the mean ± SEM of n = 4 mice per group. *P < 0.05 by the Mann–Whitney U test. (TIFF) [file ppat.1007474.s009.tiff]

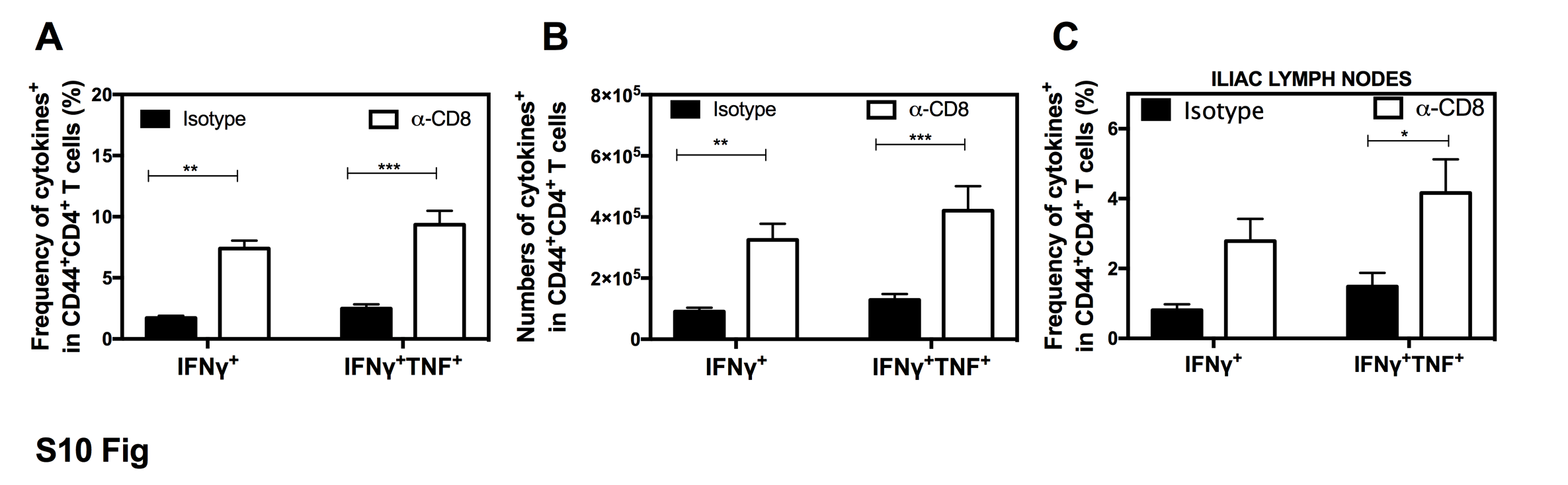

Supplement: S10 Fig — Eight-week-old LysMCre+Ifnar1fl/fl C57BL/6 mice were administered anti-CD8 (n = 8) or an isotype control Ab (n = 8) on days −3 and −1 prior to and every 2 days after intravaginal administration of 10% FBS/PBS (mock-infected) or 105 of ZIKV strain FSS13025. On day 10 post-infection, spleen and iliac lymph nodes were collected, and single-cell suspensions were stimulated in vitro with the CD4+ T cell ZIKV epitope E644-658. Cells were analyzed by flow cytometry for the (A) frequency and (B) total number of IFNγ- and IFNγ + TNF-producing CD44+CD4+ T cells in the spleen and (C) frequency of IFNγ- and IFNγ + TNF-producing CD44+CD4+ T cells in the iliac lymph nodes. Data are the mean ± SEM of n = 8 mice per group. **P < 0.01, ***P < 0.001 by the Mann–Whitney U test. (TIFF) [file ppat.1007474.s010.tiff]

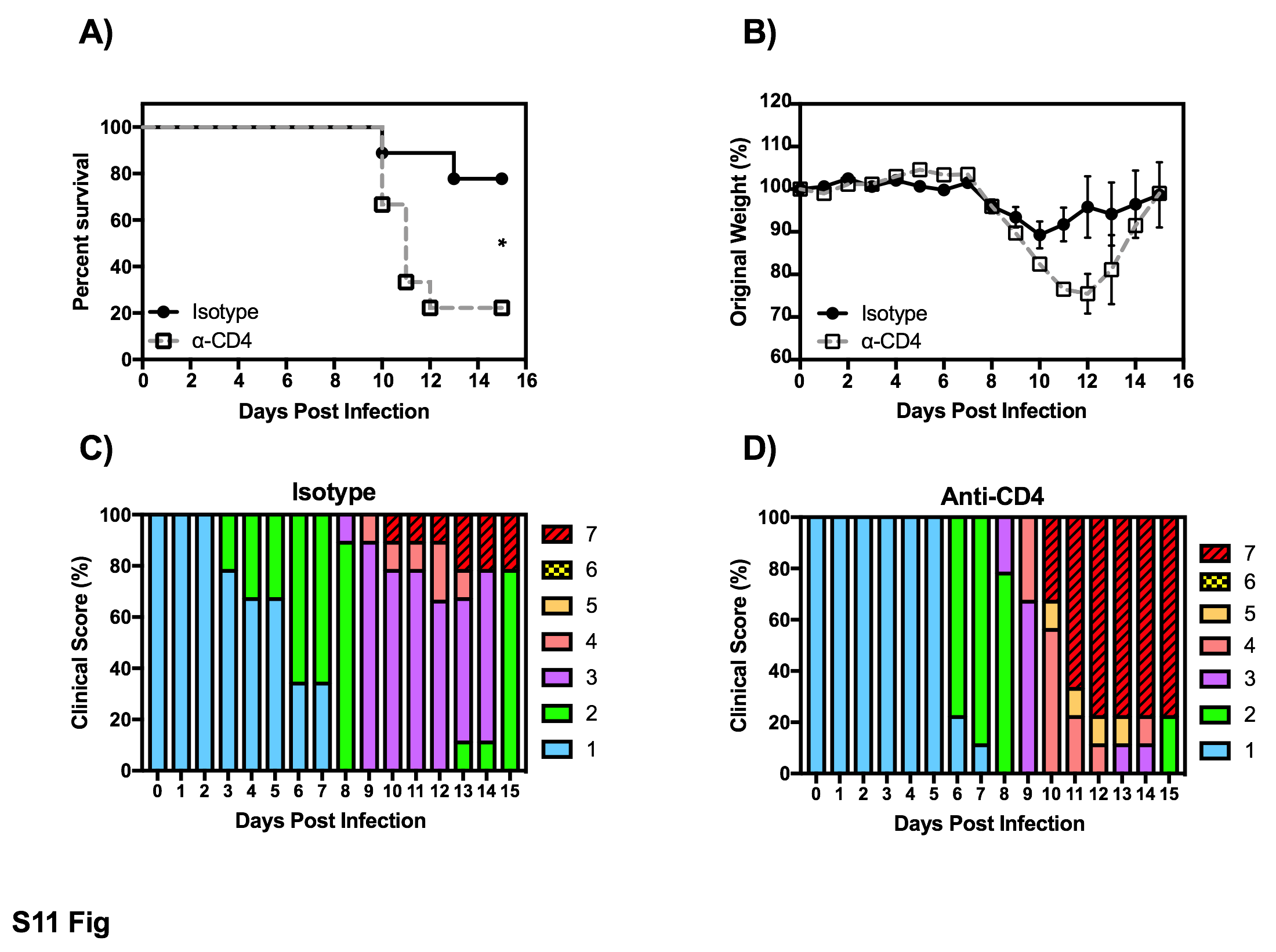

Supplement: S11 Fig — (A–D). Eight-week-old Ifnar1−/− C57BL/6 mice were treated with progesterone and anti-CD4 Ab (n = 9) or isotype control Ab (n = 9) on days −3 and −1 prior to IVag infection with 106 FFU of ZIKV FSS13025. (A) Mortality. (B) Percentage weight loss vs. day 0. (C and D) Clinical disease score for the isotype control Ab-treated group (C) and anti-CD4 Ab-treated group (D) were monitored daily and represented. Data are the mean ± SEM. **P < 0.01. Mann–Whitney U test was used to compare weight loss at each time point, and Gehan–Breslow Wilcoxon test was used to compare survival. Data were pooled from two independent experiments. (TIFF) [file ppat.1007474.s011.tiff]
